# Determining Ancestry Proportions in Complex Admixture Scenarios in South Africa Using a Novel Proxy Ancestry Selection Method

**DOI:** 10.1371/journal.pone.0073971

**Published:** 2013-09-16

**Authors:** Emile R. Chimusa, Michelle Daya, Marlo Möller, Raj Ramesar, Brenna M. Henn, Paul D. van Helden, Nicola J. Mulder, Eileen G. Hoal

**Affiliations:** 1 Computational Biology Group, Department of Clinical Laboratory Sciences, Institute of Infectious Disease and Molecular Medicine, University of Cape Town, Medical School, Cape Town, South Africa; 2 MRC Centre for Molecular and Cellular Biology, DST/NRF Centre of Excellence for Biomedical TB Research, Division of Molecular Biology and Human Genetics, Faculty of Health Sciences, Stellenbosch University, Tygerberg, South Africa; 3 MRC Human Genetics Research Unit, Division of Human Genetics, Department of Clinical Laboratory Sciences, Institute for Infectious Diseases and Molecular Medicine, Faculty of Health Sciences, University of Cape Town, Cape Town, South Africa; 4 Department of Genetics, Stanford University, Stanford, California, United States of America; 5 Department of Ecology and Evolution, Stony Brook University, Stony Brook, New York, United States of America; University of Utah, United States of America

## Abstract

Admixed populations can make an important contribution to the discovery of disease susceptibility genes if the parental populations exhibit substantial variation in susceptibility. Admixture mapping has been used successfully, but is not designed to cope with populations that have more than two or three ancestral populations. The inference of admixture proportions and local ancestry and the imputation of missing genotypes in admixed populations are crucial in both understanding variation in disease and identifying novel disease loci. These inferences make use of reference populations, and accuracy depends on the choice of ancestral populations. Using an insufficient or inaccurate ancestral panel can result in erroneously inferred ancestry and affect the detection power of GWAS and meta-analysis when using imputation. Current algorithms are inadequate for multi-way admixed populations. To address these challenges we developed PROXYANC, an approach to select the best proxy ancestral populations. From the simulation of a multi-way admixed population we demonstrate the capability and accuracy of PROXYANC and illustrate the importance of the choice of ancestry in both estimating admixture proportions and imputing missing genotypes. We applied this approach to a complex, uniquely admixed South African population. Using genome-wide SNP data from over 764 individuals, we accurately estimate the genetic contributions from the best ancestral populations: isiXhosa 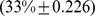, ‡Khomani SAN 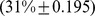, European 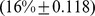, Indian 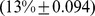, and Chinese 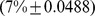. We also demonstrate that the ancestral allele frequency differences correlate with increased linkage disequilibrium in the South African population, which originates from admixture events rather than population bottlenecks.

**Nomenclature:**

The collective term for people of mixed ancestry in southern Africa is “Coloured,” and this is officially recognized in South Africa as a census term, and for self-classification. Whilst we acknowledge that some cultures may use this term in a derogatory manner, these connotations are not present in South Africa, and are certainly not intended here.

## Introduction

The field of population genetics has experienced a resurgence in the past few years due to access to extensive single nucleotide polymorphism data. The availability of genome-wide multi-locus genotype profiles has fueled long-standing interest in analyzing patterns of genetic variations to trace the ancestry components of recently admixed human populations, to identify genes underlying ethnic difference in disease risk and shed light on both the evolutionary history and migrations of recently admixed human populations [1]–[4]. In order to understand the genetic variation which could be observed at genetic marker locations within and among populations, the inference of both local ancestry and population structure from the genotypes of single nucleotide polymorphisms is crucial. These inferences, including the imputation of missing genotypes in genome-wide association studies (GWAS) utilize panels of reference ancestral populations based on place-of-origin, ethnic or continent affiliation [5]–[13]. Fortunately, the availability of high-throughput genotype data from various populations may facilitate the choice of best proxy ancestry of a recently admixed population from a pool of reference populations. This choice is critical in both the study of population genetics and in identifying genes underlying ethnic difference in genetic diseases risk [1]–[4]. Furthermore, the accuracy of these inferences is in part related to the choice of reference populations. An insufficient or inaccurate ancestral proxy can weaken these inferences, resulting in erroneous inferred ancestry, and errors and uncertainty in the imputed genotypes. These issues may consequently affect the inference of ancestry and the detection power of GWAS and meta-analysis when using imputation, particularly in multi-way admixed populations.

Because distinct populations exhibit substantial variation in genetic disease risk, the choice of reference populations for a multi-way admixed population may be sensitive and critical in biomedical research. Current algorithms for identifying the best proxy ancestral populations are inadequate for multi-way admixed populations, including HAPMIX [14], LAMPLD [5], MULTIMIX [15] and PCADMIX [16]. Furthermore, Patterson et al.(2010) utilized a regression-style technique to compute the degree of admixture given samples from an admixed population, and samples from the populations believed to be contributing. Their method was able to report on the continental admixture underlying the genetic origin of the SAC, however given an ethnic group within different populations, their method cannot tell which population is the best proxy representing the ancestral genetic donor to the gene pool of a multi-way admixed population, as was the case of the SAC in their study. In addition, the indigenous Khoesan ethnic group in southern Africa, which is well known to have historically contributed to the gene pool of the SAC, was under-represented in their study.

To address these challenges and the uncertainty in ancestral populations we developed PROXYANC, an approach to select proxy ancestry for recently admixed populations. We implemented two novel algorithms in PROXYANC, based on population genetic differentiation and optimal quadratic programming, respectively. We demonstrated through simulation of a complex multi-way admixed population that these two algorithms can select best proxy ancestry for an admixed population, given a pool of related/unrelated or admixed reference populations. Our simulation demonstrated that our complementary algorithms perform better in selecting the best proxy-ancestry for a multi-way admixed population, compared to the f3 statistic [17]. In addition we demonstrated the impact of choosing the best proxy ancestral populations in both estimating admixture proportions (global and local ancestry) and imputing missing genotypes in a multi-way admixed population, which may reduce the computational cost of the imputation processes in selecting the best haplotypes among several reference populations.

The South African Coloured population (SAC) has a high level of intercontinental admixture and therefore diverse ancestry [18]–[21]. Historical sources and a few genetic studies have reported that this population is the result of unions between African (Bantu and Khoesan), Europeans, and various other population groups of Indian or Indonesian descent [18]–[21]. A South African government source (http://www.statssa.gov.za), describes the present population of South Africa to be characterized by diversity, including groups originating from African (79%), European (9.6%) and Asian (2.5%) populations. A study conducted by Tishkoff and colleagues [18] on the characterization of the microsatellite genetic variation and the relationships among populations across the African continent, revealed that the ancestral components of the SAC include nearly equally high levels of southern African San, Niger-Kordofanian (West/Central Africa Bantu), Indian, European, and lower levels of East Asian ancestry. However, their study used 39 samples of the SAC, possibly including Cape Malays [20]. Based on 20 samples from the SAC population, a study by Patterson et al. [20] showed that there is substantial genetic contribution from at least four distinct population groups in the SAC, including Europeans, South Asians, Indonesians and a population genetically close to the isiXhosa sub-Saharan Bantu. Quintana-Murci and co-authors [21] examined the gender-specific ancestry contributions in the SAC, using mitochondrial DNA 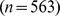 and Y-chromosome 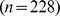 variation analysis. They inferred at least five different ancestral populations (Khoesan, Bantu, Europeans, Indians, and South-East Asians). An in-depth investigation by De Wit et al. [19] had the advantage of using a very large cohort of the SAC (959 samples) and 75,000 autosomal single nucleotide polymorphisms (SNPs) common to HapMap and Human Genome Diversity Project (HGDP) data sources. The study exploited both subsets of selected random SNPs and ancestry informative markers (AIMs) from 75,000 autosomal SNPs, to address the question of ancestry contribution in the SAC. This early investigation used a small sample of Khoesan (5 samples of San obtained from HGDP), and no suitable ancestral population samples from local southern African populations, and inferred four major contributions to the SAC with the greatest from San Africans, followed by non-San Africans, Europeans and a smaller Asian contribution [19]. However, the low San sample size may have biased the estimate of the ancestry contributions. Overall, the above investigations have documented the genome-wide continental average admixture proportions in the SAC to be in the range of 23% to 65% for African, 19% to 40% for European, and 7% to 10% for Asian, with some regional variation, and also with substantial variation among individuals. While different authors have focused on the global admixture (continental admixture) underlying the genetic origin of the SAC, attention has not yet been paid to which specific sub-continental populations or ethnic groups contributed to the admixture. In addition, recent studies demonstrated the extensive divergence between different Khoesan populations (estimated to be 30,000 years or more) and between Khoesan and Bantu-speaking groups [21]–[23]. The complexity of African population history and high differentiation between populations makes identifying the best African ancestral reference populations for the SAC crucial for local genetic ancestry inference. The greater the accuracy of the choice of ancestral population, the greater the utility in admixture mapping methods, in the imputation of missing genotypes, and in estimating global and local ancestry in multi-way admixed populations.

Here we developed PROXYANC, a novel method to select the best ancestral populations in multi-way admixed populations. We characterized the African, European, South Asian and East Asian origins of the SAC by applying PROXYANC to a cohort of the SAC (764 unrelated individuals) and report the considerable refinement of the contributions of genetic ancestry components. We establish that the SAC has had substantial admixture from isiXhosa, ‡Khomani, European, Indian (Gujarati) and Chinese populations. Using the best proxy ancestral populations found by PROXYANC, we demonstrated that the ancestral allele frequency differences correlated with increased linkage disequilibrium (LD) in the SAC, indicating that increased admixture LD is present in this population, and the observed LD has its origin from admixture events. This result supports the rejection of the hypothesis of founder effects or of population bottlenecks that could have been due to the racial segregation of the past, formalized during the recent apartheid regime in South Africa.

## Results

### Proxy Ancestral Selection

We developed the method PROXYANC, which searches for the best combination of reference populations that can minimize the genetic distance between the admixed population and all possible synthetic populations, consisting of a linear combination from reference populations (see Materials and Methods). For genetic distance, the 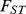 was used as an objective function of ancestral proportions as variables through an optimal quadratic cone programming algorithm. In the same vein, PROXYANC also computes a proxy-ancestry score by regressing a statistic for LD (at short distance <0.25 Morgan) between a pair of SNPs in the admixed population against a weighted ancestral allele frequency differentiation (see Materials and Methods). To evaluate PROXYANC, we mimic a 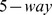 admixture scenario by simulating (see Materials and Methods) the genomes of 750 individuals of mixed ancestry through the haplotype samples from Europeans (CEU), ‡Khomani, isiXhosa, Chinese (CHD) and Indian Gujarati with probability related to our prior estimate on the ancestral proportion from each putative ancestral population (20%, 32%, 29%, 8% and 11%, respectively). We applied both approaches implemented in PROXYANC to select the best ancestral proxies for the above simulated data using 5 distinct pools from 20 reference populations that includes the African Bantu (isiXhosa, Bantu South Africa, Yoruba, Kongo, Herero), South Asian (Gujarati, Pathan, Druze), East Asian (CHD, Dai, Daur, Japanese), European (CEU, Russian, Italian, French) and Khoesan (‡Khomani, Ju|’hoan, Bushmen, SAN) populations. From each pool, our algorithms select the best ancestral populations for our simulated data. The result from the simulation demonstrates that the highest proxy-ancestry scores (Table 1) are from the five reference populations that contributed to the admixture in the simulated data (Figure S1). The higher the proxy score, the more likely it is that the related reference population is a good proxy ancestral population. In addition, among different linear combinations of five reference populations, the linear combination formed from the five populations used in our simulation (CEU, ‡Khomani, isiXhosa, Chinese and Gujarati) minimizes the genetic distance (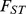) within the simulated data (Table 2). This result demonstrates that the selected proxy ancestries are in agreement and consistent with the ancestral populations used to generate these 750 admixed individuals (simulation data). To compare our algorithms to the 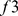 statistic [17], which is a 3-population test for admixture given two reference populations and the admixed population (target), we applied the 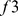 statistic to the same simulated data above, within each pair of populations from the 5 pools of reference populations described above. The results in Table 3 and Table S2 demonstrate that in many cases the 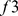 statistic fails to provide clear evidence/non-evidence of admixture in our simulated data which mimicked a multi-way admixed population. Given different pools of reference populations for a multi-way admixed population, the 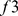 statistic clearly does not enable an accurate selection of the best proxy ancestry from each pool. Although the reference populations within a given pool may be closely related, the simulation shows that both approaches developed in PROXYANC are complementary and can select the best proxy populations that separated the closest in time from the true ancestor.

**Table 1:** Proxy Ancestry Score: results from simulation Data.

Populations	PScore	Standard Error	Z
African non-click Speaking Group			
isiXhosa	−0.124	1.138	219.793
Bantu South Africa	−0.015	0.001	28.648
Yoruba	−0.010	0.001	27.101
Kongo	−0.008	0.001	40.658
Herero	−0.008	0.001	28.306
South Asia Group			
Gujarati	0.015	0.007	223.504
Pathan	−0.007	0.001	26.427
Druze	−0.008	0.001	22.115
East Asia Group			
CHD	−0.001	0.003	118.144
Dai	−0.008	0.001	30.695
Daur	−0.007	0.001	42.628
Japanese	−0.008	0.001	26.487
European Group			
CEU	0.019	0.009	274.700
Russian	−0.008	0.001	33.347
Italian	−0.008	0.001	30.793
French	−0.008	0.001	30.716
African click-speaking Group			
‡Khomani	0.010	0.007	174.846
Ju|’hoan	−0.007	0.001	35.968
Bushmen	−0.007	0.001	34.664
SAN	−0.008	0.001	25.196

Proxy-ancestry score for 5 distinct pools, including African (isiXhosa, Bantu South Africa, Yoruba, Kongo, Herero), South Asia (Gujarati, Pathan, Druze), East Asia (CHD, Dai, Daur, Japanese), European (CEU, Russian, Italian, French) and click-speaker groups (‡Khomani, Ju|’hoan, Bushmen, SAN) using the simulated data. The results indicate that the highest scores in each pool are from CEU, ‡Khomani, isiXhosa, Chinese (CHD) and Gujarati.

**Table 2:** Top 16 linear combinations that minimize the objective function 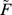 between simulated data and a combination of 5 reference populations.

Population Linear Combination	F	Standard error	95%*CI*
(isiXhosa, Gujarati, CHD, CEU, ‡Khomani)	−0.00075	0.0005599	(−0.001, 0.0005)
(isiXhosa, GIH, CHD, CEU, SAN)	−0.00058	0.0005599	(−0.001, 0.0005)
(isiXhosa, GIH, CHD, Italian, SAN)	−0.00057	0.0005599	(−0.001, 0.0005)
(isiXhosa, GIH, CHD, Italian, ‡Khomani)	−0.00054	0.0005599	(−0.001, 0.005)
(isiXhosa, GIH, Japanese, Italian, SAN)	−0.00053	0.0005586	(−0.001, 0.0005)
(isiXhosa, GIH, Japanese, Italian, ‡Khomani)	−0.00054	0.0005586	(−0.001, 0.0005)
(isiXhosa, GIH, Japanese, CEU, SAN)	−0.00051	0.0005585	(−0.001, 0.0005)
(isiXhosa, GIH, Japanese, CEU, ‡Khomani)	−0.00054	0.0005586	(−0.001, 0.0005)
(Yoruba, GIH, CHD, Italian, SAN)	−0.000371	0.0001110	(−0.0005, −0.0001)
(Yoruba, GIH, CHD, Italian, ‡Khomani)	−0.000361	0.0001110	(−0.0005, −0.0001)
(Yoruba, GIH, CHD, CEU, SAN)	−0.000371	0.0001110	(−0.0005, −0.0001)
(Yoruba, GIH, CHD, CEU, ‡Khomani)	−0.000372	0.0001110	(−0.0005, −0.0001)
(Yoruba, GIH, Japanese, Italian, SAN)	−0.000362	0.0001085	(−0.0005, −0.0001)
(Yoruba, GIH, Japanese, Italian, ‡Khomani)	−0.000365	0.0001085	(−0.0006, −0.0001)
(Yoruba, GIH, Japanese, CEU, SAN)	−0.000362	0.0001085	(−0.0005, −0.0001)
(Yoruba, GIH, Japanese, CEU, ‡Khomani)	−0.000362	0.0001085	(−0.0005, −0.0001)

The top linear combination is CEU, ‡Khomani, isiXhosa, Chinese (CHD) and Gujarati, consistent with Table 1 and with our simulation scheme.

**Table 3:** 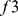
 Statistic: the signal of admixture in the simulation data.

Pop 1	Pop 2	Target	f3	Standard Error	Z
CEU	SAN	Simulated data	−0.00827	0.00149	−5.57
CEU	CHD	Simulated data	0.01321	0.00085	15.58
CEU	Gujarati	Simulated data	0.02476	0.00079	31.33
CEU	Herero	Simulated data	−0.00586	0.00140	−4.18
CEU	isiXhosa	Simulated data	−0.01748	0.00049	−36.0
CEU	‡Khomani	Simulated data	−0.0163	0.00051	−32.13
CEU	Pathan	Simulated data	−0.00602	0.00156	−3.86
CEU	Russian	Simulated data	−0.00451	0.00137	−3.29
CHD	SAN	Simulated data	−0.00289	0.00208	−1.39
CHD	Gujarati	Simulated data	0.02148	0.000794	27.134
CHD	isiXhosa	Simulated data	−0.01389	0.00057	−24.19
CHD	Italian	Simulated data	−0.00178	0.00166	−1.07
CHD	Japanese	Simulated data	−0.00352	0.00157	−2.24
CHD	‡Khomani	Simulated data	−0.01133	0.00058	−19.53
CHD	Pathan	Simulated data	−0.00308	0.00163	−1.89
CHD	Russian	Simulated data	−0.00111	0.00167	−0.7
Gujarati	isiXhosa	Simulated data	−0.01537	0.00049	−31.34
Gujarati	‡Khomani	Simulated data	−0.01452	0.00051	−28.27
‡Khomani	Druze	Simulated data	−0.00139	0.00106	−1.321
‡Khomani	French	Simulated data	−0.00151	0.00098	−1.54
‡Khomani	Herero	Simulated data	−0.00084	0.00105	−0.80
‡Khomani	isiXhosa	Simulated data	0.00247	0.00036	6.79
‡Khomani	Italian	Simulated data	−0.00128	0.00103	−1.24
‡Khomani	Japanese	Simulated data	−0.00042	0.00104	−0.40
‡Khomani	Kongo	Simulated data	−0.00076	0.00096	−0.79
‡Khomani	Pathan	Simulated data	−0.00023	0.00107	−0.22
‡Khomani	Russian	Simulated data	−0.0011	0.00097	−1.1

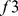
 Statistic: the signal of admixture in the simulation data (simulation obtained from 5-way admixture of ‡Khomani, isiXhosa, Chinese (CHD) Gujarati Indian and CEU) using pair-wise ancestral populations. The 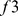 statistic fails to provide clear evidence/non-evidence of population admixture based on simulated data of 5-way admixed population.

### PROXYANC: Estimating Admixture Proportion and Imputing Missing Genotype in Admixed Populations

**Figure 1:**
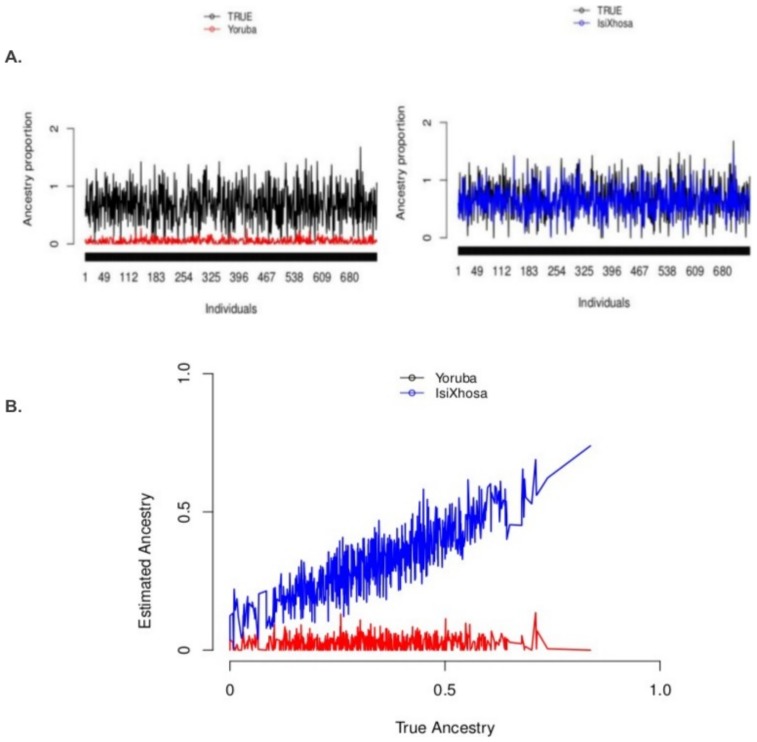
Comparison of true individual admixture proportions versus those estimated using appropriate and inappropriate proxy ancestry. (A) Plot of the estimated individual’s ancestry from best proxy ancestry (IsiXhosa:blue) and the true individual’s ancestry from the 750 admixed individuals (Black) obtained from the simulation. Plot of inappropriate proxy ancestry (Yoruba:red) estimated individual’s ancestry and the true individual’s ancestry from the 750 admixed individuals (Black) obtained from the simulation (see Materials and Methods). (B) Plot of the true ancestry versus the estimated individual’s ancestry from best proxy ancestry (IsiXhosa) and the estimated individuals ancestry from inappropriate proxy ancestry (Yoruba).

To evaluate the impact of selecting the best proxy ancestral population for an admixed population in estimating admixture proportion, we ran the ADMIXTURE software on three data sets (each of which includes the simulated admixed data set): 1) the original samples from (CEU, isiXhosa, ‡Khomani, CHD and Gujarati Indian); 2) the expanded samples from (CEU, isiXhosa, ‡Khomani, CHD and Gujarati Indian); and 3) a separate set of putatively unrelated ancestral populations (see Materials and Methods). The results from the first two panels produced the estimate of the contributions in the simulation data of the following ancestral populations; CEU:(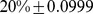 and 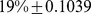), CHD:(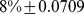 and 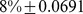), Gujarati:(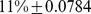 and 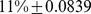), isiXhosa:(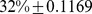 and 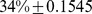) and ‡ Khomani:(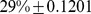 and 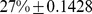), are in close agreement with the ancestry proportions used in our simulation (Figure S2). We ran ADMIXTURE again on the simulated data within a panel that included reference populations that are geographically close to the selected proxy ancestral populations, including Russian, Japanese, Palestine, Yoruba and Ju|’hoan. Compared to the true ancestral proportions used in our simulation, we obtained biased admixture proportions: 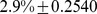 from both Russian and Palestinian, 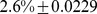 from Japanese, 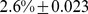 from both Yoruba and Ju
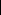
’hoan and 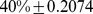 and 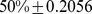 from two unknown populations (Figure S2). An example of an African ancestry case (isiXhosa versus Yoruba contribution in the simulated data) is displayed in Figure 1. In Figure 1, we compared the true individual admixture proportions versus those estimated from the best proxy ancestry (isiXhosa) and an inappropriate proxy ancestry (Yoruba), respectively. The estimated individual admixture proportions from isiXhosa are closer to the true individual ancestral proportions compared to proportions from Yoruba (Figure 1). This result shows the importance of selecting the best proxy ancestry in estimating admixture proportions.

**Figure 2:**
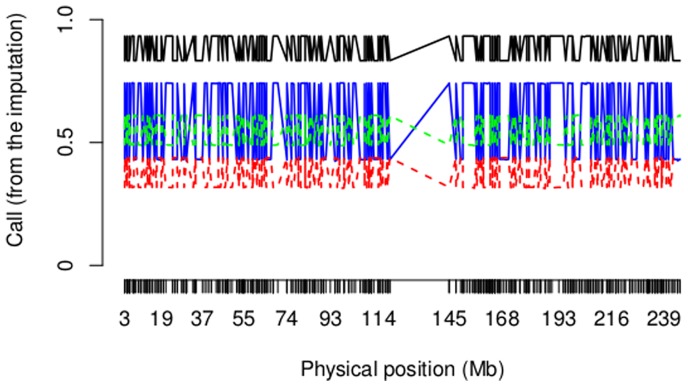
Figure 2. Genotype call rate when imputing missing genotypes for the simulated data. 2044 SNPs were imputed for the simulated data using 4 sets of reference populations. Panels included Black: (CEU, CHD, GIH, isiXhosa, ‡Khomani), Green: (Initial samples from CEU, CHD,GIH, isiXhosa, ‡Khomani), Blue: All populations used to evaluate PROXYANC (see Materials and Methods) and Red: (Russia, Japanese, Palestine, Yoruba and Ju|’hoan). Using a panel of best proxy ancestral populations of multi-way admixed population can produce as similar accurate results of the imputation of missing genotype as using all available reference populations, and highlights the benefit of using correct proxy ancestral populations through the imputation of missing genotype in multi-way admixed populations which may reduce the computational cost of the imputation engine to choose the best haplotype among several available reference populations.

Including all available reference populations in imputing has recently been discussed to be useful in accurately imputing genotypes. However, it becomes computationally expensive to the imputation engine to choose the best haplotype among several available reference populations [24], [25]. To address this, we assess whether a panel of selected best proxy ancestral populations can achieve similar accuracy as using all available reference populations, in imputing missing genotypes of an admixed population. We removed 2044 SNPs out of 39064 SNPs on chromosome 1 from the simulated data, and we imputed them using 4 different sets of reference populations. These four sets of reference populations included a panel of populations (CEU, CHD, Gujarati, isiXhosa, ‡Khomani) used directly in the simulation (that have equal sample sizes of 1500 each, see Materials and Methods), a panel of populations (CEU, CHD, Gujarati, isiXhosa, ‡Khomani) used to test PROXYANC (see Materials and Methods), a panel of all 20 populations listed in the 5 pools described in Results, and a panel formed by the Russian, Japanese, Palestinian, Yoruban and Ju|’hoan populations. The result in Figure 2 indicates a high call rate when imputing missing genotypes of the simulated data using the correct proxy ancestries. The imputation using the first panel of populations used directly in our simulation, converged to perfectly imputed genotypes. The imputation using the second and third panels also yielded accurately imputed genotypes. Due to the small sample size used in the second panel (Material and Methods), the imputation based on this panel (consisting of five proxy ancestral populations (see Table 1) with their original sample size) has a lower genotype call rate compared to the first panel. Using the last panel of populations, which does not include proxy ancestors, we obtained poor imputation (Figure 2).

### PROXYANC: Selecting Proxy Ancestry in the SAC

To select the best proxy ancestral populations using the real SAC data, we applied PROXYANC to 5 pools of reference populations implicated by both PCA and admixture analysis. We first constructed African, European, South Asian and East Asian population data sets using populations described in Table S1, each including 764 unrelated SAC samples. The data analyzed was from four sources: The African population panels [26] (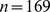 samples from 11 African populations genotyped on an Illumina Beadchip 550*K* custom *v*2 chip and Affymetrix 500*K*), the Human Genome Diversity Cell Line Panel [27] (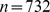 samples from 53 populations genotyped on an Illumina 650*K* array), the International Haplotype Map (HapMap) Phase 3 [28] (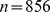 samples from 10 populations genotyped on an Illumina 1*M* array), and samples from 6 southern African populations obtained from Pickrell et al. [22], Henn et al. [16], [26] and Schlebusch et al. [23] (Table S1). We performed admixture analysis using the ADMIXTURE software [11] and Principal Component Analysis (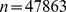 autosomal SNPs) on each data set described above. We were able to identify the candidate reference populations for the proxy ancestry analysis (Figures S3–S8). We performed both proxy ancestry score and 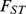-optimal quadratic cone programming on 5 distinct pools of reference populations. The results from both proxy ancestry score (Table 4 and Figure S9) and 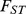-optimal quadratic cone programming (Table 5) were in agreement and reveal that the combination of CEU, isiXhosa, Gujarati, CHD, and ‡Khomani formed the best proxy ancestry for the SAC (Table 4 and Table 5). This result suggests a Southern Bantu population (isiXhosa), and South African San (‡Khomani) to be the best Bantu and San proxy ancestral population for the SAC, compared to the more frequently used Yoruba and the Namibian San of previous studies [18], [19], [21].

**Table 4:** Proxy Ancestry Score: results from the South African Coloured.

Populations	PScore	Standard Error	Z
South Asia Group			
Kalash	−0.003	0.001	1483.76
Gujarati	0.003	0.001	2224.43
Pathan	−0.002	0.001	1511.30
African Non-Click Speaking Group			
Fulani	0.001	0.002	1822.48
Bantu South Africa	0.001	0.001	1822.48
Yoruba	0.004	0.001	2282.03
Tswana	0.003	0.001	2237.05
isiXhosa	0.003	0.001	2350.63
Bamoun	−0.002	0.001	1769.27
Brong	0.001	0.001	2013.24
Herero	0.002	0.001	2180.48
African Click-speak Group			
SAN	0.002	0.001	2150.70
Hadza	−0.003	0.001	1783.85
Sandawe	0.001	0.001	2064.319
Bushmen	−0.003	0.001	1784.10
Ju|’hoan	0.003	0.002	2206.76
‡Khomani	0.007	0.001	2612.07
East Asia Group			
She	−0.007	0.001	1181.64
Dai	−0.003	0.001	1579.25
Daur	−0.004	0.001	1329.53
CHB	−0.003	0.001	1523.72
CHD	−0.003	0.001	1544.38
Japanese	−0.003	0.001	1443.25
European Group			
Sardinia	−0.003	0.001	1463.5
Belgarmo	−0.001	0.001	1668.56
CEU	0.000	0.001	1891.314
Russian	−0.002	0.001	1535.53
French	−0.001	0.001	1723.62

Proxy-ancestry score for 5 distinct pools, including African non-click speaking group, East Asian, European, click-speaking group and South Asian populations using the SAC data. The result shows that the highest scores are from CEU, ‡Khomani, isiXhosa, Chinese and Gujarati in the relevant pool.

**Table 5:** 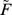
 as an Objective Function: Results from South African Coloured Data.

Pop Linear Combination	F	Standard error	95%*CI*
(Gujarati, Sotho, ‡Khomani, CHB, CEU)	0.0042	0.0010	(−0.006, −0.0025)
(Gujarati, Sotho, ‡Khomani, CHB, Russian)	−0.0042	0.00102	(−0.006, −0.0023)
(Gujarati, Sotho, ‡Khomani, CHD, CEU)	−0.0042	0.00101	(−0.006, −0.0023)
(Gujarati, Sotho, ‡Khomani, CHD, Russian)	−0.0042	0.00101	(−0.006, −0.0023)
(Gujarati, isiXhosa, ‡Khomani, CHB, CEU)	−0.00374	0.00060	(−0.005, −0.003)
(Gujarati, isiXhosa, ‡Khomani, CHB, Russian)	−0.00374	0.00060	(−0.005, −0.003)
(Gujarati, isiXhosa, ‡Khomani, CHD, CEU)*	−0.00374	0.00060	(−0.005, −0.003)
(Gujarati, isiXhosa, ‡Khomani, CHD, Russian)	−0.00374	0.00060	(−0.005,−0.003)
(Gujarati, Brong, ‡Khomani, CHB, CEU)	−0.02483	0.00605	(−0.037, −0.013)
(Gujarati, Brong, ‡Khomani, CHB, Russian)	−0.02483	0.00605	(−0.037, −0.013)
(Gujarati, Brong, ‡Khomani, CHD, CEU)	−0.02483	0.00605	(−0.037, −0.013)
(Gujarati, Brong, ‡Khomani, CHD, Russian)	−0.02483	0.00605	(−0.037, −0.013)

Top 12 linear combinations that minimize the objective function 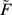 between SAC data and a combination of 5 pools of reference populations. The top linear combination is CEU, ‡Khomani, isiXhosa, Chinese (CHD) and Gujarati, consistent with Table 4.

### Refinement of Admixture Proportions in the SAC

**Figure 3:**
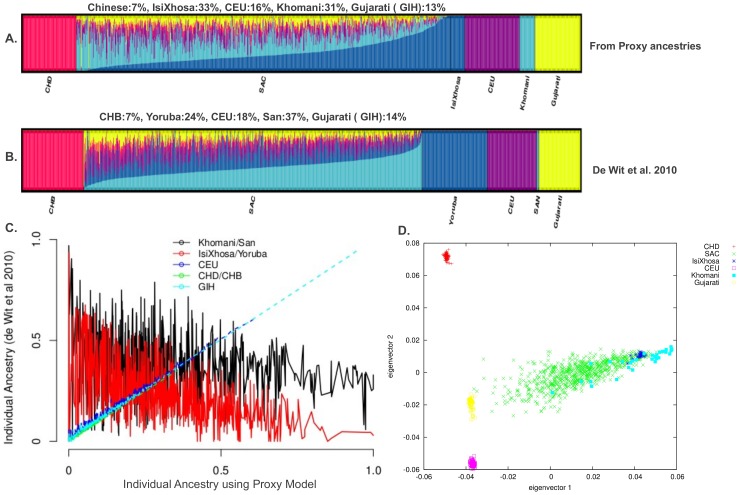
Individual ancestry proportion and PCA based on 47863 autosomal SNPs in the SAC data. (A) Population clustering analysis of the SAC using both the current selected best proxy ancestors as reference panel (First top figure) and reference panel used in De Wit et al.(B) Plot showing individual’s ancestry difference between panel of selected best proxy ancestral population of the SAC and the panel of reference population used in De Wit et al. [19]. This plot indicates a large difference of African ancestry of the SAC between the two analyses, suggesting the choice of African Ancestry of the SAC is critical and sensitive due to the diversity and closely relatedness of most African populations. (C) PCA on autosomal SNPS. Both first and second principal components show great genetic differentiation between the five proxy ancestral groups, where the SAC is in the convex hull of them.

Using the result from PROXYANC on the SAC data, we combined the top proxy ancestral populations (CEU, CHD, Gujarati, isiXhosa, ‡Khomani) (Tables 4 and 5), including the SAC, in one data set. We repeated both the PCA and the ancestral population clustering analysis. From these analyses, our inferred five major ancestral contributions (Table 6 and Figures 3 (A–B)) to the SAC population have a roughly equivalent African ancestral proportion from isiXhosa (33%) and ‡Khomani (31%), followed by European (CEU) (16%), Gujarati Indian (12%) and a smaller admixture proportion from the Chinese (8%). It is also clear from the PCA plots in Figure 3(D), that the SAC lie on a direct line with these five groups of proxy ancestors. In addition, both isiXhosa and ‡Khomani groups were related to the SAC, indicating their close ancestral affiliations with this population, and this reflects the role of both Southern Bantu and indigenous Khoesan south of the Kalahari in the early establishment of the SAC population [29]. The other putative groups of proxy ancestral populations, CEU, Gujarati Indian and Chinese, are separated from each other, and the SAC is in the convex hull of the three. These findings agree well with the result obtained from the admixture analysis on 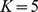 in Figure 3(A–B). As we expected, the PCA in Figure 3 (D) revealed the greatest genetic differentiation between these five proxy ancestries of the SAC, which clearly reflects the admixture of the SAC from these five proxy ancestors. In addition, we compared our estimated admixture proportions with previous estimates in Patterson et al. [20], and we redid the admixture analysis using the ancestral populations used in De Wit et al. [19] that included the Yoruba, CEU, San, Gujarati, and Chinese (CHB). Figure 3 (B) indicates a large difference in African ancestry of the SAC between the two analyses (using the proxy ancestries panel and the panel from De wit et al. 2010), suggesting that the choice of African ancestry for the SAC is critical when conducting ancestry inferences and admixture mapping studies. This may be due to the diversity and close relatedness of most African populations. Table 6 displays the estimated admixture proportions from our selected best proxy ancestries and from those two previous studies. Our result highlights the importance of selecting the best proxy ancestral populations for multi-way admixed populations, and we demonstrate that inaccurate proxy ancestries can result in inaccurate inferred ancestry which is fundamental to admixture association or admixture mapping studies, and can therefore lead to erroneous interpretation of results identifying genomic location underlying genetic ancestry difference in complex diseases risk.

**Table 6:** Summary mean and standard error on proportion of ancestral populations contributing to the genetic make-up of the South African Coloureds.

This Study				
**isiXhosa**	**‡Khomani**	**CEU**	**CHD**	**Gujarati**
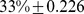	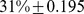	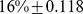	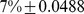	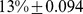
**De Wit et al.** [**19**]				
**Yoruba**	**SAN**	**CEU**	**CHB**	**Gujarati**
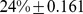	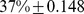	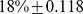	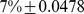	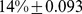
**Reported ancestral proportions in Patterson et al. 2009**				
**isiXhosa**	**X**	**European**	**Indonesian**	**South Asian**
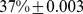	–	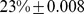	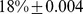	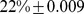

This table displays the mean and the standard errors of ancestral proportions with the best proxy ancestors obtained from PROXYANC, with the reference populations panel used in De Wit et al. [19] and the SAC’s ancestral proportions reported in Patterson et al. [20].

Taken together, the results above provide confidence that our inferred five ancestral components with balanced African contributions from isiXhosa and ‡Khomani populations, followed by Northern and Western European, Gujarati Indian and a smaller Chinese contribution, is closer to the true picture of ancestral contributions according to the SAC’s history. We believe that our result also has the advantage of handling sample size differences and using accurate proxy ancestral populations, and believe that both the number of SNPs (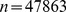 ) and target population sample size used can provide sufficient resolution to support our inferred ancestral contribution.

### Linkage Disequilibrium and Genetic Diversity

Understanding the extent of admixture LD is useful in designing disease mapping tests in admixed populations [2], [30]. To assess the pattern of admixture LD in the SAC as a result of ancestral admixture, we first compared LD between the SAC and its putative proxy ancestors (see Materials and Methods). We calculated the LD (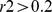) across the whole genome of each population and found that the LD is consistently higher at very short distances in the SAC (Figure S10). The LD in the SAC decays from regions >0.2 Morgan (Figure S10), suggesting that this LD is primarily a result of admixture rather than founder effects. This finding is consistent with prior studies that established that the admixture LD decays within a few generations at long distances (>20 cM) but decays slowly at short distances (<10 cM) [31], [32]. Recent admixture between genetically differentiated populations gives rise to an increase in admixture LD proportions [2], [30]. To test for the admixture LD due to these five proxy ancestral population admixtures in the SAC, we computed the LD between all pairs of markers in the SAC, weighted by their frequency difference (see Materials and Methods) between each pair of these five proxy ancestral populations, isiXhosa and ‡Khomani, European (CEU), Gujarati Indian and Chinese(CHD). Through linear regression of the allele frequency differences of each pair of proxy ancestral groups with LD in the SAC, we obtained a correlation (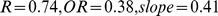) with a significant p-value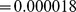, indicating an association of allele frequency differences with increased LD in the SAC. We finally estimated the maximum expected admixture LD (see Materials and Methods) from each pair of proxy ancestral populations. We compared this with the observed LD in the SAC. Table 7 shows that the correlation between the expected admixture LD from each pair of proxy ancestral groups and the observed LD in the SAC is significant, except for CEU-GIH, and CEU-CHD, which may be due to the fact that the GIH and CHD proportions are small (Figure S11). Through an additive linear model, we obtained a lower p-value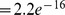 under the null hypothesis of no correlation between LD in the SAC and these maximum expected admixture LD, indicating that the LD in the SAC correlated with the expected admixture LD, and mainly has its origin in admixture between the populations related to these five proxy ancestries. This result confirms that admixture between populations related to these five proxy ancestral groups (isiXhosa and ‡Khomani, European (CEU), Gujarati Indian and Chinese (CHD)) largely contributed to the admixture LD observed in the present SAC population.

**Table 7:** Correlation between maximum expected admixture LD and the observed LD in the SAC.

Pair-wise populations	P-value	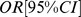
(CHD, Gujarati)	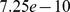	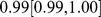
(isiXhosa, Gujarati)	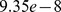	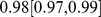
(CEU, CHD)	0.92	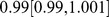
(CHD, ‡Khomani)	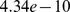	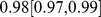
(‡Khomani, isiXhosa)	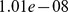	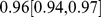
(‡Khomani, Gujarati)	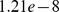	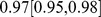
(CEU, Gujarati)	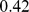	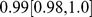
(CEU, 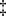 Khomani)	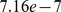	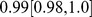
(CHD, isiXhosa)	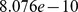	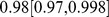
(CEU, isiXhosa)	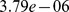	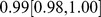

P-value obtained from the correlation between expected admixture LD from each pair of proxy ancestral group with respect to the observed LD in the SAC.

We additionally compared the genome-wide haplotype diversity and the percentage haplotype sharing by IBD (see Materials and Methods), and the result in Table S3 indicates that the SAC has a higher haplotype diversity than any of its five proxy ancestral groups. The result suggests that both the higher diversity and higher LD at short distances observed in the SAC are the result of admixture events, and not founder effects or an extreme bottleneck. The observed higher level of genetic diversity in the admixed SAC is likely to be the result of the geographic location of South Africa with respect to major trade routes in the past (from the 15th to the 19*th* centuries) and its history of multi-faceted colonization [29].

## Discussion

We introduce PROXYANC, an approach to select proxy ancestry for complex multi-way admixed populations. We assessed its accuracy through a simulation of a multi-way admixed population and demonstrated the impact and sensitivity of the choice of reference panel in estimating global and local ancestry and in imputing missing genotypes. Because of increased urban development and migration, the proportion of individuals with significant recent genetic admixture is on the increase in many modern societies. However, increased population admixture influences genome heterozygosity, which in turn will affect phenotypes relevant to health. Therefore, the choice of the best proxy ancestral populations for an admixed population is critical in both the study of population genetics and in identifying genes underlying ethnic differences in genetic disease risk. Our simulation results demonstrated the usefulness of the choice of proxy ancestry for admixed populations, in contributing to the accuracy of the inferences of both local and global ancestry. Selecting an accurate proxy ancestral population for an admixed population is required for improving the power of GWAS for admixed populations. Furthermore, our simulation demonstrated that the proxy ancestral panel achieved a similar accuracy to that including all available populations in imputing missing genotypes of an admixed population. This indicates that the choice of accurate ancestral panel can help in reducing computational costs of the imputation engine for finding the best haplotype among all available populations during imputation processes.

To the best of our knowledge, PROXYANC is the first approach to select the best reference ancestral panel given pools of reference ancestral panels. Our methods to select proxy ancestral populations in a multi-way admixed population have enabled us to characterize the genetic ancestry component of the uniquely admixed Coloured population of South Africa that accounts for 49% of the population of the Western Cape Province of South Africa (Statistics South Africa, Census 2011). Previous studies of this historically complex population were hampered by the relatively small sample size and few putative ancestral populations publicly available, and especially the very low number of San individuals. In the present study we have utilized the increased number of reference populations available, and the best proxy ancestries of the SAC obtained from PROXYANC allowed us to document a contribution of the isiXhosa, ‡Khomani, European, Gujarati Indian, and Chinese genetic material to the SAC (33%, 31%, 16%, 12% and 7%, respectively). We expected a southern Bantu-speaking group such as isiXhosa instead of a West African group such as the Yoruba to be a better proxy ancestor of the SAC. The isiXhosa as best proxy ancestor of the SAC reflects the early mixing of mainly indigenous San females with the southern Bantu groups and potentially the distinct genetic profile of southern Bantu-speaking populations after their expansion through Africa. Subsequently male settlers, mainly from the Netherlands, Britain, Germany and France, and male slaves from South Asia [29], [33], [34] also contributed to the SAC. The substantial number of ‡Khomani (southern Kalahari San) individuals available for this study greatly increases our confidence in the accuracy of the ancestry estimates presented here. Our results also emphasize the point that San clans are often very different from one another, and grouping San individuals from different areas together as generic “San” may result in a loss of discrimination at the genetic level [21], [22]. This was also illustrated by the deep genetic differences between individual San (Bushmen) genomes [35]. In the case of the SAC in the Western Cape, it is perhaps to be expected that San groups from the southern Kalahari including ‡Khomani, which is geographically closer to the place of origin of the SAC, to be a better proxy ancestors of this group than Ju|’hoan from Namibia, and this is what we have shown (Table 4). This also gives credence to an earlier suggestion that only some of the San populations contributed to the SAC population [21].

A higher degree of LD is expected in admixed populations, and this could at certain points of its history be influenced by population bottlenecks, or still be a result of the admixture itself. We demonstrated that the allele frequency differences between each pair of proxy ancestral populations correlated with the degree of LD in the SAC, suggesting that the admixture increased genetic diversity and that the observed LD in the SAC has its origin mainly in the admixture. This study observed a weak level of founder haplotypes identical-by-descent along the genome of the SAC, which strengthens the evidence against the argument that past legislated separation of ethnic groups in South Africa, including the SAC, caused population bottlenecks. However in spite of this isolation, the original admixed population was large and a population bottleneck is therefore unlikely.

The obtained best proxy ancestry for the SAC provides opportunities to examine an accurate, unbiased estimation of the ancestry at each genetic locus in this multi-way admixed population, to potentially provide crucial insights into identifying disease genes based on ethnic difference. As existing methods that infer local ancestry assume that non-admixed ancestral populations are the most suitable, it may not be advisable to use the isiXhosa, which have some Khoe-San ancestry [36] as an ancestral population for admixture mapping. Until such time as these methods are updated, the highest ranking putative non-admixed African Bantu populations listed in Table 4, such as the Yoruba, can be used as proxy ancestral population(s) instead of the isiXhosa.

In conclusion, this study has highlighted the importance of selecting the best proxy ancestry for potential downstream analysis in a multi-way admixed population. The investigation of admixture LD and the identification of source populations for the SAC has not only deepened our understanding of its evolutionary history, but also provide opportunities for designing a method to account for a combined genome-wide SNP case-control and admixture mapping in a multi-way admixed population such as the SAC. PROXYANC will also provide a useful tool for the investigation of other multi-way admixed populations.

## Materials and Methods

### Ethics Statement

Approval from the Ethics Committee of the Faculty of Health Sciences, Stellenbosch University (project registration numbers 95/072 and 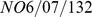) was obtained before blood samples for DNA were collected with written informed consent. This research was conducted according to the principles expressed in the Declaration of Helsinki.

### Genotype Data and Genotype Quality Control

The DNA samples of 764 unrelated individuals who self-identified as South African Coloured (SAC) from two suburbs of Cape Town were collected and genotyped using the Affymetrix 500*K* genotyping platform, as described in De Wit et al [19]. A total of 159 samples from southern African populations obtained from Pickrell et al. [22], Henn et al. [26], Schlebusch et al. [23] and (HGDP-CEPH) [27] were used in this study. Additionally, we incorporated genome-wide SNP data from two public data sources, including the Human Genome Diversity Cell Line Panel (HGDP-CEPH) [27], the International Haplotype Map (HapMap) Phase 3 [28]. Detailed information about the number of individuals included in our analysis is provided in Table S1. We performed quality-control filters on each population separately and removed SNPs that failed the Hardy-Weinberg exact test P<0.000001 and had a call rate <95% across all samples per population using PLINK [37]. Population outliers and unknown relatedness were assessed using the smart program implemented in EIGENSOFT [10], [38] and related samples were excluded. After applying the quality-control filters to each population separately, the SNPs genotyped in this study were reduced to a subset (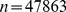) shared between the SAC, the three public data sources and the local southern Bantu from South Africa (Table S1). Grouping each population per continent, we were able to construct African, European, South Asian, East Asian, Middle East, American and Oceania data sets, each including the SAC.

### Mathematical Details of PROXYANC

The question we want to address is, given a pool of available continental affiliated populations, for example European or African, which population is the best European or African proxy ancestry of the admixed population under study. We assume prior knowledge of geographical potential ancestral populations.

### PROXYANC: 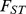-optimal Quadratic Cone Programming

To limit the effect of background linkage disequilibrium, let us assume adjacent SNPs in each population that are spaced 10 Kb from each other. Let Z denote a set of pools of distinct reference ancestral populations. Let 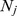 and 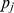 be the total variant allele count and observed population allele-frequency in the admixed population (*A*), 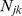 and 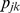 be the total variant allele count and the population observed allele-frequency in a particular reference population 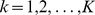 of unrelated individuals at SNP 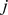. Given different combinations C of 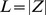 reference populations of unrelated individuals from each pool 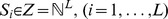, each combination 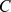 of 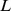 reference populations can be obtained from the Cartesian product 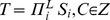. Thus, from each 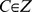 we construct synthetic populations consisting of 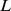 populations as the following linear combination,
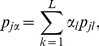
(1)where 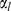 is the ancestral proportion. A particular combination of 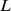 populations consists of best proxy ancestries of *A* if their linear combination (in equation 1) minimizes a constructed (equation 2) objective function 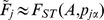. 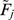 is approximated from a classical 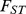 function in order to render the optimization problem convex. The model described in here is related to optimal quadratic cone programming, where the objective function 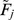 is given by,
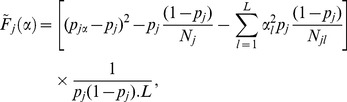
(2)at SNP 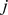, subject to 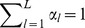 and



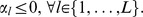



Equation 2 is a generalized objective function such as that described in Price et al. (2009a) [6] and is a quadratic convex function with respect to 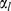 (ancestry proportion, 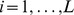), therefore a global minimum can be found. Expanding and rearranging equation 2 (see File S1), we obtain a matrix representation of the optimal Cone Programming of the form,
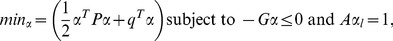
(3)where 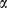 is a vector of L-dimensions of unknown ancestry proportions, 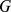 is an identity vector of L-dimensions, 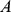 is a vector of allele frequencies of L-dimensions, 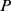 is a positive semi definite matrice, and its diagonal elements are all coefficients of the quadratic term,
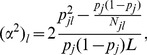
(4)and the mixture coefficients 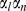 consist of its symmetric elements, and are given by:
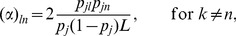
(5)and the linear coefficients 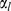 are the elements of vector 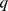 in equation 3, and are represented by:



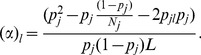
(6)For the optimization of equations (3) or (2) with respect to 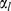 (ancestry proportions, 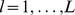), the matrix form in equation (3) is constructed by summing equations (2), (4), (5) and (6) respectively across all SNPs. The details of the above model can be found in the File S1.

### PROXYANC: Proxy-Ancestry Score

When admixture occurs between two or more previously isolated populations with differences in allele frequency, admixture creates linkage disequilibrium (LD) between genetic loci. Accounting for this assumption, we can compute the proxy ancestry score from the data of the admixed population and pair-wise reference populations. Computing the correlation between the LD in the admixed population and allele frequency differentiation in each pair of ancestral populations, the Proxy-Ancestry Score algorithm is as follows:

1. Given 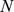 samples from the data of the admixed population and the data of 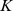 groups of reference populations without missing genotypes data, we compute the expected squared correlation 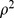 between each pair of SNPs 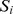 and 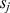, 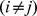 in the data of the admixed population.

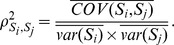



Taking the Fisher’s transformation on 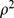,
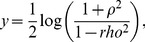
(7)thus, we compute the LD for each pair of SNPs located at distances (<0.2 Morgans),



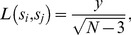
(8)(2) For each different pair of reference populations, we compute the variant allele frequency difference 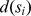 and 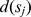 at the corresponding SNP 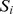 and 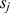, 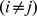 used in the previous step. Assuming two ancestral populations 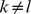, where 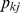 and 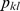 are variant allele-frequencies in 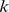 and 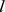 respectively,



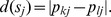

1. We regress 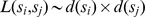, and obtain a p-value, 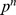, 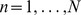.
2. For 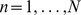 possible combinations of each reference population 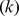 with other reference ancestral populations, we compute the inverse normal distribution 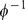
on the p-value resulting from step (3) as

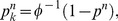
(9)In this way, a smaller p-value corresponds to a larger 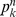.

1. For each reference population 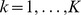, we compute the proxy ancestry score,

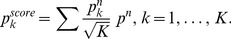
(10)(6) To determine whether the proxy ancestry score in equation 14 is higher than expected, we normalized it. To address this we consider a vector of all proxy ancestry scores.



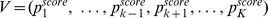
excluding 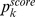, and we compute the normalization of it as follows,



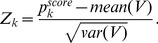
(11)All the methods above have been implemented in a python module called PROXYANC (http://www.cbio.uct.ac.za/proxyanc).

### Simulation Framework to Test PROXYANC

To start our simulation, we independently phased each putative ancestral population, including European (CEU), isiXhosa, ‡Khomani, East Asia (CHD) and Gujarati Indian using BEAGLE [39]. To generate 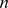 diploid admixed individuals, our simulation framework uses 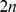 ancestral haplotypes, where 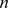 should be the minimum sample size among the parental populations. Therefore, we independently expanded each putative ancestral population following Rogers and Harpendings (1992) model of exponential population growth. We implemented this model using three parameters, 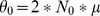, 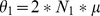 and 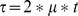, where an initial population of effective size 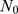, is assumed to grow exponentially to a new size of 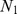 at a time 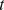 generations back from the present. The mutation rate 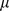, is the per-generation probability that a mutation strikes a random nucleotide along the genome. Therefore, each ancestral population was expanded to a total size of 1500 plus its original size. We split the resulting samples in two separate groups. 1500 samples from each of these expanded reference populations were used to simulate admixed individuals and the remaining samples were set aside. The original population samples were used to test PROXYANC.

To simulate the genome of an admixed individual that can mimics the genetic make-up of a multi-way admixed population such as the SAC, we sample haplotypes from European (CEU), isiXhosa, ‡Khomani, East Asia (CHD) and Gujarati Indian with probability related to our prior estimate of the ancestral proportion from each putative ancestral population (20%, 32%, 29%, 8% and 11%, respectively). Considering a continuous gene flow model [14] in 100 generations and accounting for the Wright-Fisher model with random mating, from the beginning to the end of each chromosome, the ancestry is re-sampled using related ancestral proportion above, at each SNP in order to identify the occurrence of the admixture event. Following this process, the chromosomal segment of ancestral population is copied to the genome of the admixed individual, and records the locus-specific ancestry (the true ancestry) which will serve to assess the estimated ancestry. Using this procedure, we simulated the genomes of 750 individuals of mixed ancestry from Europeans (CEU), isiXhosa, ‡Khomani, East Asia (CHD) and Gujarati Indian.

To evaluate PROXYANC, we applied both approaches implemented in PROXYANC (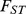-optimal quadratic cone programming and proxy-ancestry score) to select the best ancestral proxy for the above simulated data. Since the true number of ancestral populations is known, one can choose closely related or geographically close populations to the true ancestral populations. In real data, where the number of ancestral populations is unknown, it is necessary to do a pre-population structure using ADMIXTURE [11], for example. Here, we use a pool of 20 reference populations geographically close to the true ancestral populations, including CEU, Italian, French, Russian, Gujarati, Pathan, Druze, isiXhosa, Bantu South Africa, Herero, Kongo, Yoruba, ‡Khomani, Ju|’hoan, SAN, Bushmen, dai, Chinese(CHD) Japanese (JPT) and Daur. Particularly, for these five putative ancestral populations (CEU, isiXhosa, ‡Khomani, East CHD and Gujarati), we used the initial samples that were not used in either expansion or the simulation of the admixed population in order to avoid overestimate.

To assess the impact of selecting the best reference ancestral populations in estimating the admixture proportions, we separately ran the ADMIXTURE software [11] on the simulated data together with the expanded and initial samples from ancestral populations (CEU, isiXhosa, ‡Khomani, CHD and Gujarati Indian), respectively (as described above). We also ran ADMIXTURE on the simulated data together with a panel that included reference populations that are geographically close to the selected proxy ancestral populations, including Russian, Japanese, Palestine, Yoruba and Ju|’hoan. This allowed us to assess the estimated admixture proportions with a mis-identified source population versus the true proportions.

To investigate if a restricted panel of only the selected best proxy ancestral populations of an admixed population is useful in imputing accurate genotypes (one could use all available reference populations), we removed 2,044 out of 39,064 SNPs on chromosome 1 from the simulated data, and we imputed them using 4 different sets of reference populations, including a panel of populations (CEU, CHD, GIH, isiXhosa, ‡Khomani) used directly in the simulation, a panel formed of initial samples from ancestral populations (CEU, CHD, GIH, isiXhosa, ‡Khomani) used to test PROXYANC, a panel of all 20 populations listed above and a panel formed by inappropriate proxy ancestral populations, including Russian, Japanese, Palestinian, Yoruba and Ju|’hoan populations. his allowed us to assess the imputation accuracy rate using different reference panels.

### Admixture Estimation and Principle Component Analysis of the SAC

We applied the clustering algorithm implemented in ADMIXTURE [11] to determine the ancestral population clustering on each continental data set (African, European, South Asian, East Asia, Middle East, American and Oceania, see populations in Table S1) merged separately with the SAC data. Subsequently, the best proxy ancestral populations were merged with the SAC data for supervised clustering. Averaging the SAC individual admixture proportions, we obtained the population admixture proportions (ancestry contributions). The DISTRUCT program [40] was applied on Q-matrices. In order to perform principal component analysis (PCA) to evaluate the extent of substructure of the South African Coloured population, the smartpca programme in the EIGENSOFT package [10], [38] was applied on the data sets described above.

### Admixture Linkage Disequilibrium

Increased LD in a population relative to its ancestral population(s) can be due to founder events or population bottlenecks [41]. To determine whether the SAC has undergone an extreme bottleneck, we compared the significance level of increase in LD at short distances (<0.1 cM) and long distances (>0.2 cM), within and between the SAC and its proxy ancestors. To account for the sample size effect in computing the LD, we first scaled each population sample, including the SAC sample, to roughly equal size. The LD-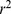 values were computed for all SNP-pairs along the genome. Thus, we directly compared the LD-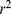 for each SNP-pair by ranking the number of pairs that had higher LD-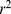 (>0.5) in the SAC to that in each proxy ancestral population. Furthermore, we computed the correlation of inter-proxy ancestral allele-frequency differences and LD-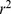 in the SAC. The allele-frequency differences were calculated on the first (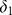) and second (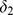) SNP based on the pair of SNPs having LD-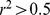 in the SAC. The correlations were then computed between 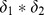 and LD-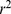 in the SAC. We reported on the average p-values and the correlations. To see whether the level of the observed admixture in the SAC can account for the increased LD, we also estimated the maximum expected admixture LD from each pair of reference ancestral populations and compared them with the observed LD in the SAC. Given the LD and allele-frequency from a pair of unrelated ancestral populations X and Y of the admixed population Z, the admixture LD metric 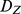 is related to the LD 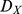 and 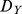 from X and Y [41], [42], and is modelled as,
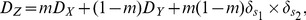
(12)at SNPS, 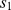 and 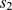, where 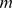 is the ancestral proportion. This equation is a quadratic equation of the second order of the form 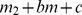, where 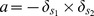, 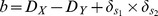 and 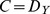. We denoted 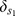 and 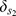 as the difference in allele frequency at genetic marker 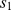 and 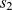 from 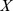 and 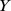 populations. To obtain the admixture proportion 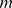 at which admixture LD reaches its maximum, we differentiate 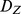 with respect to 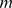 and obtain the maximum expected admixture LD as



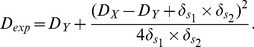
(13)To assess the admixture LD, we compute the expected square correlation between the observed LD in a recently admixed population and 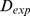 from each pair of candidate proxy ancestral populations. The above described method is also implemented in PROXYANC (http://www.cbio.uct.ac.za/proxyanc).

### Genetic Diversity and Haplotypes Shared IBD in the SAC

In order to compare the level of admixture in the SAC, we computed the proportion of IBD and the pairwise population concordance (PPC) test. For the pairwise identity-by-state (IBS) test, we ran PLINK with 10,000 permutations between populations in the same data set (SAC versus each proxy ancestral population). We coded the SAC as cases and its proxy ancestries as controls. We calculated the empirical p-values to determine whether case/case-pairs were less similar to each other compared to control/control-pairs [37], [43]. To compare the haplotypes shared IBD within and between the SAC and its proxy ancestral populations, the PLINK software package was used for this purpose. In addition, we computed the haplotype frequency from PLINK, for each population, we estimated the haplotype diversity as
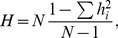
(14)where 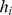 is the haplotype frequency and 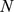 is the haplotype sample size. The mean haplotype diversity was reported. The haplotype frequency was computed for each population using PLINK.

## Supporting Information

Figure S1 **Plot of proxy-ancestry scores of each population in each group of reference populations (Subjects and Methods).** All the highest peaks can be observed from the five ancestral populations that contributed to the admixture in the simulated data.
(TIFF)

Figure S2 **Plot of individuals ancestry.** The first two plots are based on the reference populations used to simulate the admixed population and to assess PROXYANC (see Materials and Methods), respectively. The mean and its standard deviation from both analyses yielded to CEU (20%0.0999 and 19%0.1039), CHD (8%0.0709 and 8%0.0691), Gujarati (11%0.0784 and 11%0.0839), IsiXhosa (32%0.1169 and 34%0.1545) and ‡Khomani (29%0.1201 and 27%0.1428), respectively. The bottom plot is based on populations geographically close to the best proxy ancestors of the simulation data. The admixture proportion in the plot is inconsistent to the true admixture proportions in our simulated data, 2.9% from both Russian and Palestine, 2.6% from Japanese, 2.6% from both Yoruba and Ju|’hoan and 40% and 50% from two unknown populations.
(TIFF)

Figure S3 **Ancestral population clustering (A) and Principal Component Analysis (B) of the SAC and African populations.** (A) The plot in (A) is the proportion of each individual’s ancestry. (B) The plot is of the first and the second eigenvectors in the PCA of the combined populations. For clarity, the population labels in figure (A) are ordered as Kaba, Fang, MKK, ban, XHS, XHS2, LWK, SBAN, HER, STS, ASW, SAC, SAN, BUS, KHS, KHO, HAZ, SAW, Mada, man, Brong, yor, Igbo, Fulani, YRI, Hausa, Kongo, Bulala, Bamoun, TNS, moz, MRC_*S*, ALG, MRC_*N*, EGT, LBY and SAH_*occ* (Table S1).
(TIFF)

Figure S4 **Ancestral population clustering (A) and Principal Component Analysis (B) of the SAC and European populations.** (A) The plot in (A) is the proportion of each individual’s ancestry. (B) The plot is of the first and the second eigenvectors in the PCA of the combined populations. For clarity, the population labels in figure (A) are ordered as bas, fre, ita, CEU, SAC, sar, rus, ady and orc (Table S1).
(TIFF)

Figure S5 **Ancestral population clustering (A) and Principal Component Analysis (B) of the SAC and South Asian populations.** (A) The plot in (A) is the proportion of each individual’s ancestry. (B) The plot is of the first and the second eigenvectors in the PCA of the combined populations. For clarity, the population labels in figure (A) are ordered as pat, pim, SAC, sin, bur, haz, bal, kal, mak, bra and GIH (Table S1).
(TIFF)

Figure S6 **Ancestral population clustering (A) and Principal Component Analysis (B) of the SAC and East Asian populations.** (A) The plot in (A) is the proportion of each individual’s ancestry. (B) The plot is of the first and the second eigenvectors in the PCA of the combined populations. For clarity, the population labels in figure (A) are ordered as yi, CHB, hez, dau, oro, SAC, dai, tuu, mia, tuj, she, yak, CHD, cam, JPT, jap and mon (Table S1).
(TIFF)

Figure S7 **Principal Component Analysis (PCA) of the SAC and the World-wide populations (Table S1).** The first and the second eigenvectors in the PCA of the combined SAC and worldwide populations are shown.
(TIFF)

Figure S8 **Principal Component Analysis of the SAC and both American and Middle-east populations, respectively. (Table S1).** (A) The first and the second eigenvectors in the PCA of the combined SAC and American populations. (B) The first and the second eigenvectors in the PCA of the combined SAC and Middle-east populations. Both figures in (A) and (B) show no evidence of relatedness between the SAC and populations from America and Middle-east.
(TIFF)

Figure S9 **Plot of proxy-ancestry scores (Subjects and Methods) of each population in each group of reference populations.** The highest peaks indicates the best proxy ancestry for the South African Coloured population.
(TIFF)

Figure S10
**LD across all the autosomes in the SAC compared with proxy ancestral groups.** (A–E). Plots of R-square (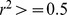) between pairs of SNPs (combined linked and unlinked SNPs) within 10 Kb from each other. In the figure, we denote ‡Khomani, CEU, CHD+Gujarati Indian, IsiXhosa and Yoruba as Khoesan, European, South-East Asian, South-African Bantu and African Niger Bantu populations, respectively.(TIFF)

Figure S11 **LD due to proxy ancestral population (CEU, ‡Khomani, CHD, Gujarati and IsiXhosa) admixture in the SAC.** To generate these plots, we computed the LD between all pairs of markers in the SAC and the expected admixture from each pair of ancestral populations. The figure is the scatter plots of LD in the SAC and the expected admixture LD in pairs of ancestral populations.
(TIFF)

Table S1 Putative ancestral populations included in the fine characterization of the South African Coloureds (SAC) and simulation data.
(PDF)

Table S2 f3 Statistic: the signal of admixture in the simulation data (simulation obtained from 5-way admixture of ‡Khomani, IsiXhosa, Chinese (CHD) and Indian Gujarati and CEU) using pair-wise ancestral populations. The f3 statistic fails to provide clear evidence/non-evidence of population admixture based on simulated data of a 5-way admixed population.
(PDF)

Table S3 Comparing genetic diversity between the South African Coloured population (SAC) and the five proxy ancestral groups contributing to the SAC admixture. Mean and standard error of shared haplotype segment in cM (Hap.segment), mean and standard error of haplotype diversity measure (Hap.diversity) and proportion of IBD (Prop.IBD).
(PDF)

File S1
**PROXYANC: 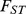-optimal Quadratic Cone Programming.**
(PDF)

## References

[1] Zhu X, Tang H, Risch N (2008) Admixture mapping and the role of population structure for localizing disease genes. Adv Genet 60, 547–69. 10.1016/S0065-2660(07)00419-1 18358332

[2] Winkler C, Nelson G, Smith M (2010) Admixture mapping comes of age. Ann Rev Genomics Hum Genet 11, 65–89. 10.1146/annurev-genom-082509-141523 PMC7454031 20594047

[3] Seldin M (2007) Admixture mapping as a tool in gene discovery. Genet Dev 17: 177–181. 10.1016/j.gde.2007.03.002 PMC3146309 17466511

[4] McKeigue P (1997) Mapping genes underlying ethnic differences in disease risk by linkage disequilibrium in recently admixed populations. Am J Hum Genet 60: 188–196. 8981962 PMC1712571

[5] Baran Y, Bogdan P, Sankararaman S, Dara G, Gignoux C, et al.. (2012) Fast and accurate inference of local ancestry in latino populations. Bioinformatics 28, 1359–1367. 10.1093/bioinformatics/bts144 PMC3348558 22495753

[6] Price A, Helgason A, Palsson S, Stefansson H, Clair D, et al.. (2009) The impact of divergence time on the nature of population structure: An example from Iceland. PLoS Genet 5(6), e1000505. 10.1371/journal.pgen.1000505 PMC2684636 19503599

[7] Pasaniuc B, Sankararaman S, Kimmel G, Halperin E (2009) Inference of locus-specific ancestry in closely related population. Bioinformatics 25, i213–i221. 10.1093/bioinformatics/btp197 PMC2687951 19477991

[8] Sankararaman S, Kimmel G, Halperin E, Jordan M (2008) On the inference of ancestries in admixed populations. Genome Res 18(4), 668–675. 10.1101/gr.072751.107 PMC2279254 18353809

[9] Tang H, Choudhry S, Mei R, Morgan M, Rodriguez-Cintron W, et al.. (2007) Recent genetic selection in the ancestral admixture of Puerto Ricans. Am J Hum Genet 81, 626–633. 10.1086/520769 PMC1950843 17701908

[10] Price A, Patterson N, Plenge R, Weinblatt M, Shadick N, et al.. (2006) Principal components analysis corrects for stratification in genome-wide association studies. Nature Genet 38, 904–909. 10.1038/ng1847 16862161

[11] Alexander D, Novembre J, Lange L (2009) Fast model-based estimation of ancestry in unrelated individuals. Genome Research 19, 1655–1664. 10.1101/gr.094052.109 PMC2752134 19648217

[12] Hoggart C, Parra E, Shriver M, Bonilla C, Kittles R, et al (2003) Control of confounding of genetic associations in stratified populations. Am J Hum Genet 72: 1492–1504. 12817591 10.1086/375613 PMC1180309

[13] Falush D, Stephens A, Pritchard J (2003) Inference of population structure: Extensions to linked loci and correlated allele frequencies. Am J Hum Genet 164, 1567–1587. 10.1093/genetics/164.4.1567 PMC1462648 12930761

[14] Price A, Tandon A, Patterson N, Barnes K, Rafaels N, et al.. (2009) Sensitive detection of chromosomal segments of distinct ancestry in admixed populations. Plos Genet 5, e1000519. 10.1371/journal.pgen.1000519 PMC2689842 19543370

[15] Churchhouse C, Marchini J (2012) Multiway admixture deconvolution using phased or unphased ancestral panels. Genet Epidemiology 37, 1–12. 10.1002/gepi.21692 23136122

[16] Henn B, Botigue L, Gravel S, Wang W, Brisbin A, et al.. (2012) Genomic ancestry of north Africans supports back-to-Africa migrations. Nat Comm 3 (1143) 2140. 10.1371/journal.pgen.1002397 PMC3257290 22253600

[17] Patterson N, Moorjani P, Luo Y, et al.. (2012) Ancient admixture in human history. Genet Society of Am 10, 112145037. 10.1534/genetics.112.145037 PMC3522152 22960212

[18] Tishkoff S, Reed F, Friendlaender F, Ehret C, Ranciaro A (2009) The genetic structure and history of Africans and African Americans. Sciences 324, 1035–1044. 10.1126/science.1172257 PMC2947357 19407144

[19] deWit E, Delport W, Chimusa R, Meintjes A, Moller M, et al.. (2010) Genome-wide analysis of the structure of the south African Coloured population in the western Cape. Hum Genet 128, 15–53. 10.1007/s00439-010-0836-1 20490549

[20] Patterson N, Petersen D, vanderRoss R, Sudoyo H, Glashoff R, et al.. (2010) Genetic structure of a unique admixed population: implications for medical research. Hum Mol Genet 19, 411–419. 10.1093/hmg/ddp505 19892779

[21] Quintana-Murci L, Harmant C, Quach H, Balanovsky O, Bormans Z, et al.. (2010) Strong maternal Khoesan contribution to the south African Coloured population: A case of gender-biased admixture. Am Soc of Hum Genet 86, 611–620. 10.1016/j.ajhg.2010.02.014 PMC2850426 20346436

[22] Pickrell K, Patterson N, Barbieri C, Berthold F, Gerlach L, et al.. (2012) The genetic prehistory of southern Africa. Nature Communications 3 (1143). doi:101038/ncomms2140. 10.1038/ncomms2140 PMC3493647 23072811

[23] Schlebusch C, Skoglund P, Sjodin P, Gattepaille L, Hernandez D, et al.. (2012) Genomic variation in seven Khoe-San groups reveals adaptation and complex African history. Science 338, 374–379. 10.1126/science.1227721 PMC8978294 22997136

[24] Marchini J, Howie B (2008) Comparing algorithms for genotype imputation. Am J Hum Genet 83, 535–539. 10.1016/j.ajhg.2008.09.007 PMC2561929 18940314

[25] Li J, Guo Y, Pei Y, Hong-Wen D (2012) The impact of imputation on meta-analysis of genome-wide association studies. PLoS ONE 7(4), e34486. 10.1371/journal.pone.0034486 PMC3320624 22496814

[26] Henn B, Gignouxb C, Jobinc M, Grankae J, Macphersonf, etal. (2011) Hunter-gatherer genomic diversity suggests a southern African origin for modern humans. PNAS 108, 5154–5162. 10.1073/pnas.1017511108 PMC3069156 21383195

[27] Cann H, de Toma C, Cazes L, Legrand M, Morel V, et al.. (2002) A human genome diversity cell line panel. Science 296, 261–262. 10.1126/science.296.5566.261b 11954565

[28] Frazer K, et al.. (2007) A second generation human haplotype map of over 3.1 million SNPs. Nature 449, 851–861. 10.1038/nature06258 PMC2689609 17943122

[29] Mountain A (2003) The first people of the Cape: A look at their history and the impact of colonialism on the Cape’s indigenous people. Cape Town, South Africa: David Philips Publishers. ISBN:0-86486-23-2.

[30] Pfaff CL, Parra EJ, Bonilla C, Hiester K, McKeigue PM, et al (2001) Population structure in admixed populations: effect of admixture dynamics on the pattern of linkage disequilibrium. Am J Hum Genet 68: 198–207. 11112661 10.1086/316935 PMC1234913

[31] Chakravati R, Weiss K (1998) Admixture as a tool for finding linked genes and detecting that difference from allelic association between loci. Proc Nat Acad Science 85, 9119–9123. 10.1073/pnas.85.23.9119 PMC282675 3194414

[32] Smith MW, O’Brien SJ (2005) Mapping by admixture linkage disequilibrium: advances, limitations and guidelines. Nature Rev Genet 6, 623–632. 10.1038/nrg1657 16012528

[33] Keegan T (1996) Colonial south Africa and the origins of the racial order. Claremont, South Africa: David Philip Publishers.

[34] Boonzaaier E, Malherbe C, Smith A, Berens P (1996) The Cape Herders: A history of the Khoikhoi of southern Africa. Cape Town: David Philip publishers.

[35] Schuster S, Miller W, Ratan A, Tomsho L, Giardine B, et al.. (2010) Complete Khoisan and Bantu genomes from southern Africa. Nature, 463, 943–947. 10.1038/nature08795 PMC3890430 20164927

[36] Nurse G, Weiner J, Jenkins T (1985) The peoples of southern Africa and their affinities. Oxford: Clarendon Press. ISBN: 0-19-857541-6.

[37] Purcell S, Neale B, Todd-Brown K, Thomas L, Ferreira M, et al.. (2007) Plink: a tool set for whole-genome association and population-based linkage analyses. Am J Hum Genet 81, 559–575. 10.1086/519795 PMC1950838 17701901

[38] Patterson N, Price A, Reich D (2006) Population structure and eigenanlysis. PLoS Genet 2(12), e190. 10.1371/journal.pgen.0020190 PMC1713260 17194218

[39] Browning BL, Browning SR (2009) A unified approach to genotype imputation and haplotype-phase inference for large data sets of trios and unrelated individuals. Pam J Hum Genet 84, 210–223. 10.1016/j.ajhg.2009.01.005 PMC2668004 19200528

[40] Rosenberg N (2004) Distruct: a program for the graphical display of population structure. Molecular Ecology Notes 4, 137–138.

[41] Kruglyak L (1999) Prospects for whole-genome linkage disequilibrium mapping of common disease genes. Nat Genet 22, 139–144. 10.1038/9642 10369254

[42] Shiheng T, Rongmei Z, Jianhua C, Xiaoming L, Liping D, et al.. (2001) A population genetics model of linkage disequilibrium in admixed populations. Chinese Science Bullin 46, 193–197.

[43] Bray S, Mulle J, Dodd A, Pulver A, Wooding S, et al (2010) Signatures of founder effects, admixture, and selection in the ashkenazi jewish population. Proc Nat Ac Sci 107: 162–16227. 10.1073/pnas.1004381107 PMC2941333 20798349

