# Supplementary figures and images for: Determining Ancestry Proportions in Complex Admixture Scenarios in South Africa Using a Novel Proxy Ancestry Selection Method
Source: PLoS One. 2013 Sep 16;8(9):e73971. doi: 10.1371/journal.pone.0073971 (PMC3774743; doi:10.1371/journal.pone.0073971)

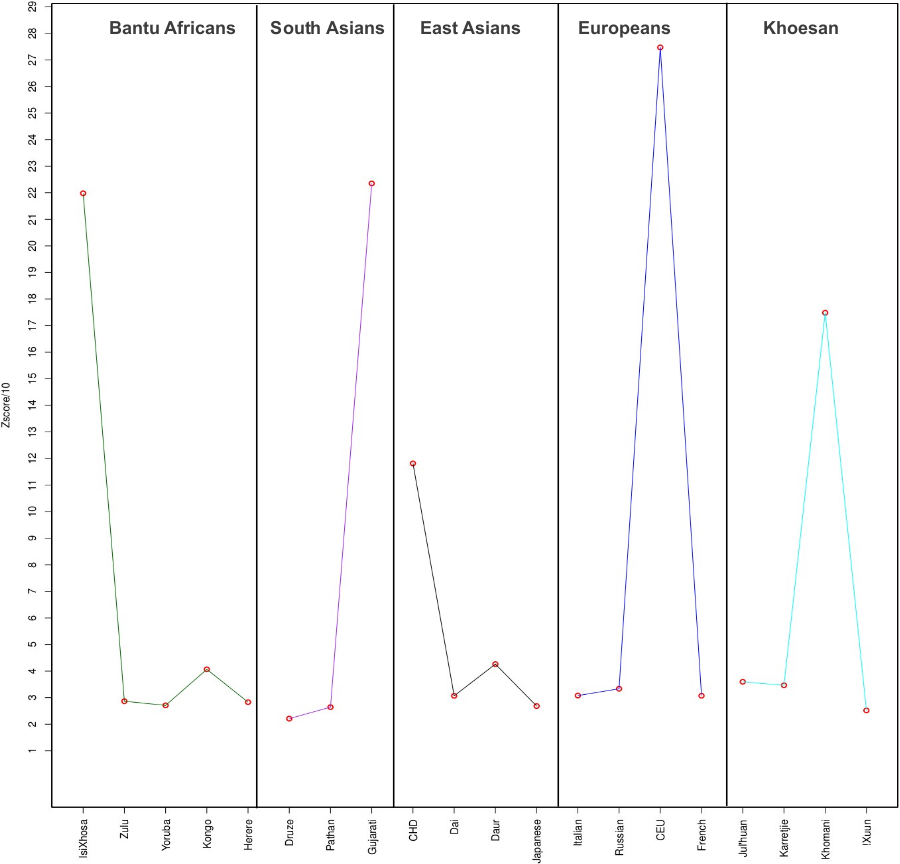

Supplement: Figure S1 — Plot of proxy-ancestry scores of each population in each group of reference populations (Subjects and Methods). All the highest peaks can be observed from the five ancestral populations that contributed to the admixture in the simulated data. (TIFF) [file pone.0073971.s001.tiff]

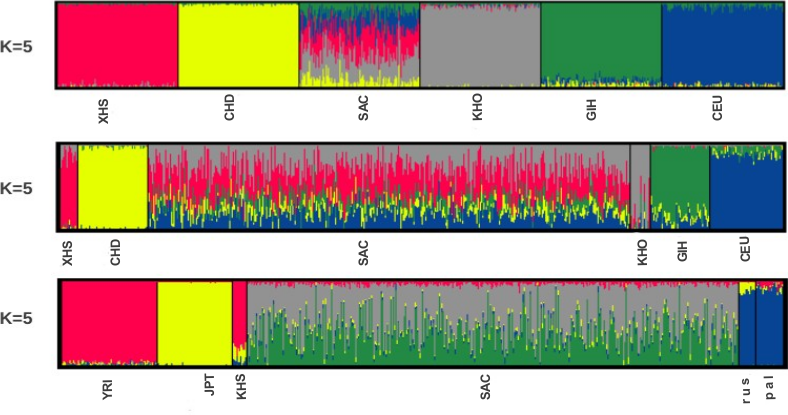

Supplement: Figure S2 — Plot of individuals ancestry. The first two plots are based on the reference populations used to simulate the admixed population and to assess PROXYANC (see Materials and Methods), respectively. The mean and its standard deviation from both analyses yielded to CEU (20%0.0999 and 19%0.1039), CHD (8%0.0709 and 8%0.0691), Gujarati (11%0.0784 and 11%0.0839), IsiXhosa (32%0.1169 and 34%0.1545) and ‡Khomani (29%0.1201 and 27%0.1428), respectively. The bottom plot is based on populations geographically close to the best proxy ancestors of the simulation data. The admixture proportion in the plot is inconsistent to the true admixture proportions in our simulated data, 2.9% from both Russian and Palestine, 2.6% from Japanese, 2.6% from both Yoruba and Ju|’hoan and 40% and 50% from two unknown populations. (TIFF) [file pone.0073971.s002.tiff]

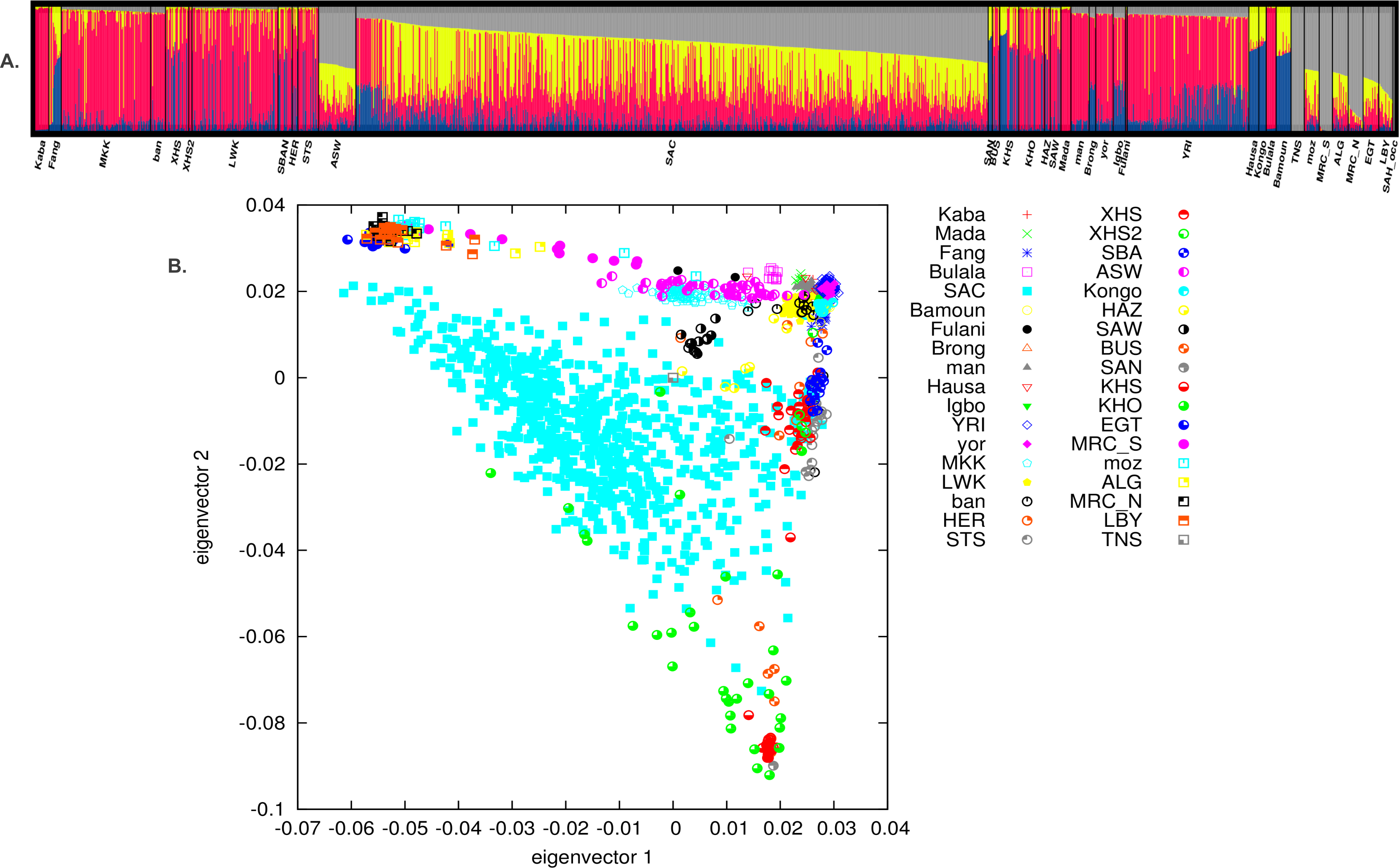

Supplement: Figure S3 — Ancestral population clustering (A) and Principal Component Analysis (B) of the SAC and African populations. (A) The plot in (A) is the proportion of each individual’s ancestry. (B) The plot is of the first and the second eigenvectors in the PCA of the combined populations. For clarity, the population labels in figure (A) are ordered as Kaba, Fang, MKK, ban, XHS, XHS2, LWK, SBAN, HER, STS, ASW, SAC, SAN, BUS, KHS, KHO, HAZ, SAW, Mada, man, Brong, yor, Igbo, Fulani, YRI, Hausa, Kongo, Bulala, Bamoun, TNS, moz, MRC_S, ALG, MRC_N, EGT, LBY and SAH_occ (Table S1). (TIFF) [file pone.0073971.s003.tiff]

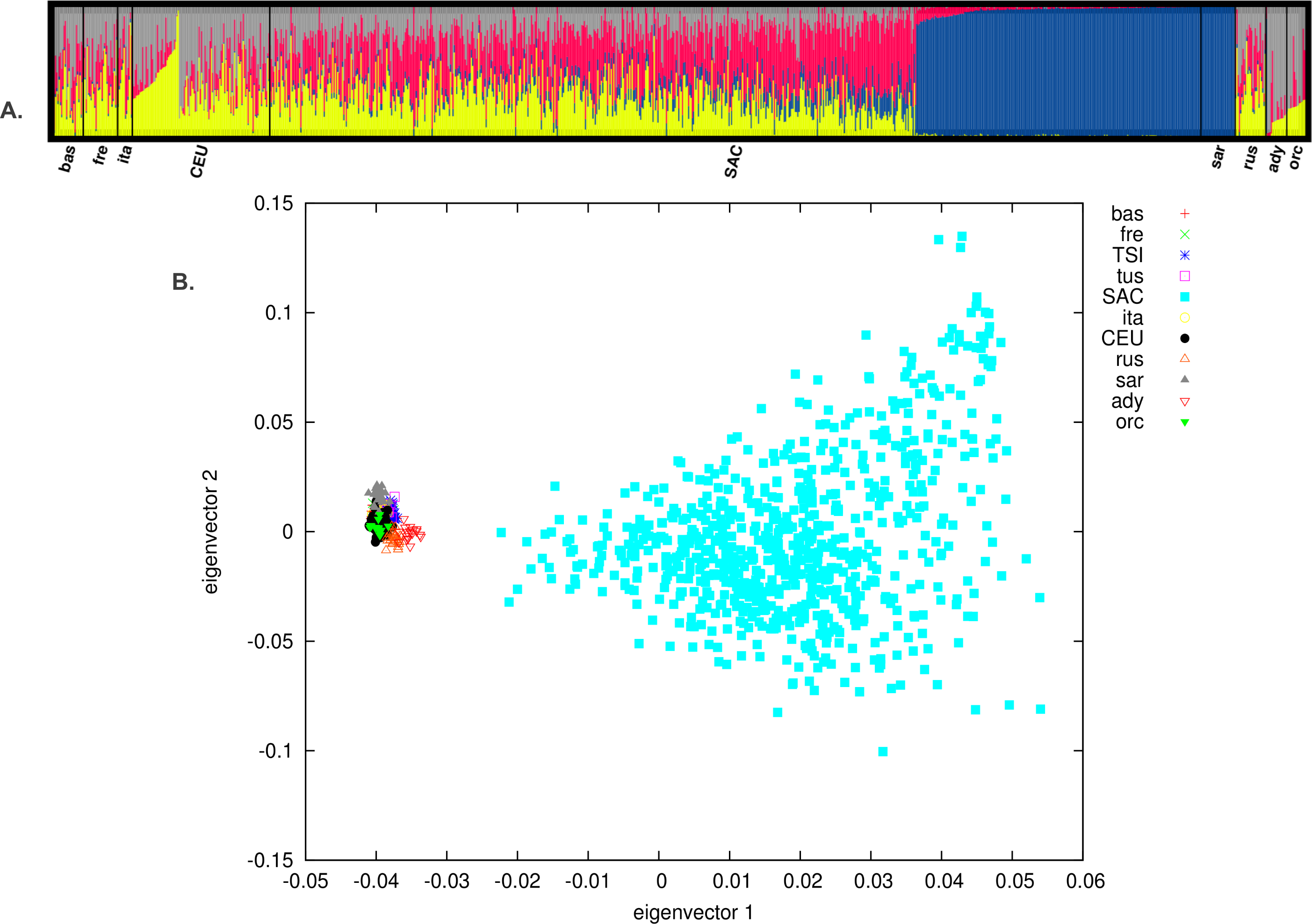

Supplement: Figure S4 — Ancestral population clustering (A) and Principal Component Analysis (B) of the SAC and European populations. (A) The plot in (A) is the proportion of each individual’s ancestry. (B) The plot is of the first and the second eigenvectors in the PCA of the combined populations. For clarity, the population labels in figure (A) are ordered as bas, fre, ita, CEU, SAC, sar, rus, ady and orc (Table S1). (TIFF) [file pone.0073971.s004.tiff]

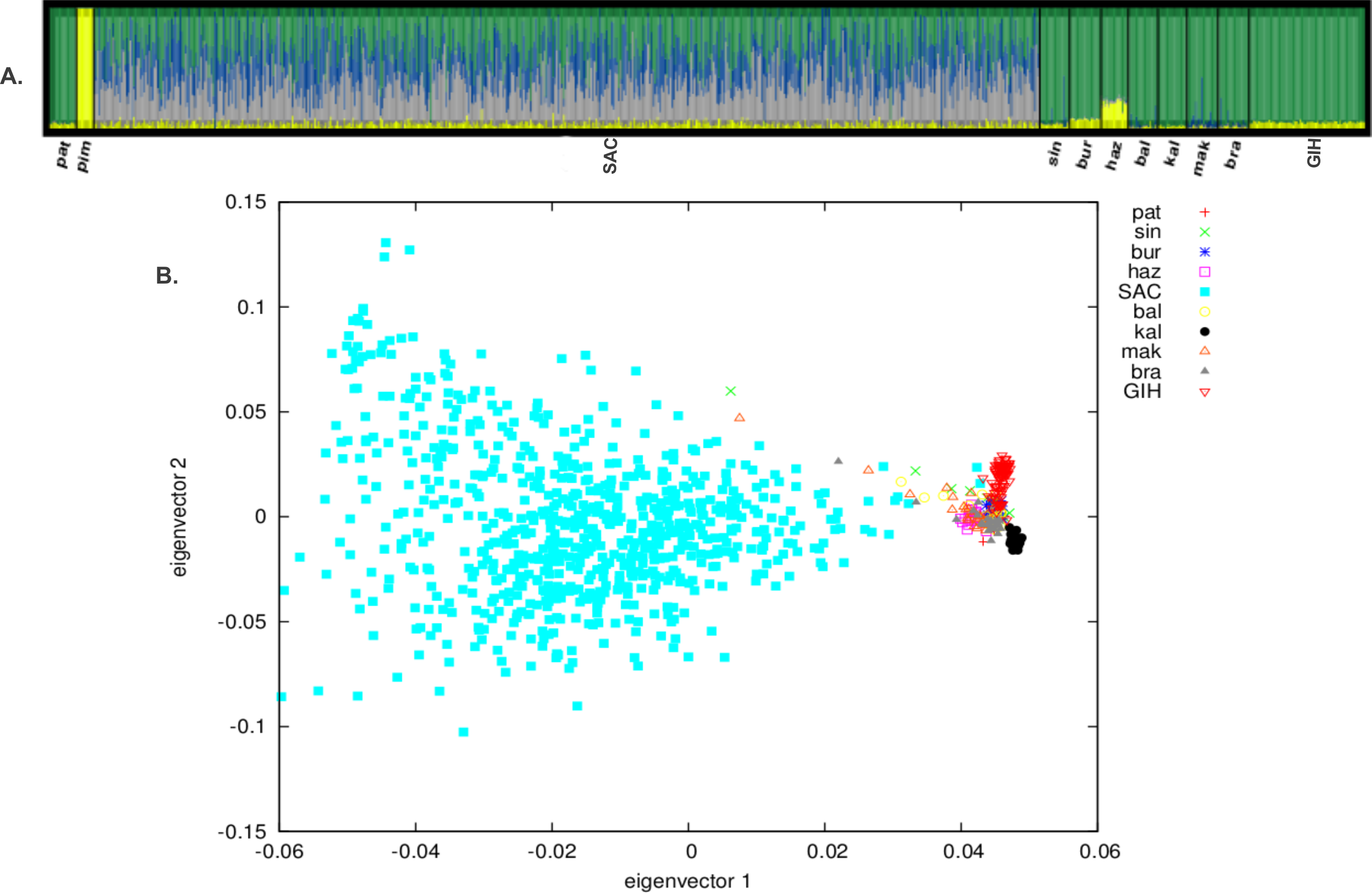

Supplement: Figure S5 — Ancestral population clustering (A) and Principal Component Analysis (B) of the SAC and South Asian populations. (A) The plot in (A) is the proportion of each individual’s ancestry. (B) The plot is of the first and the second eigenvectors in the PCA of the combined populations. For clarity, the population labels in figure (A) are ordered as pat, pim, SAC, sin, bur, haz, bal, kal, mak, bra and GIH (Table S1). (TIFF) [file pone.0073971.s005.tiff]

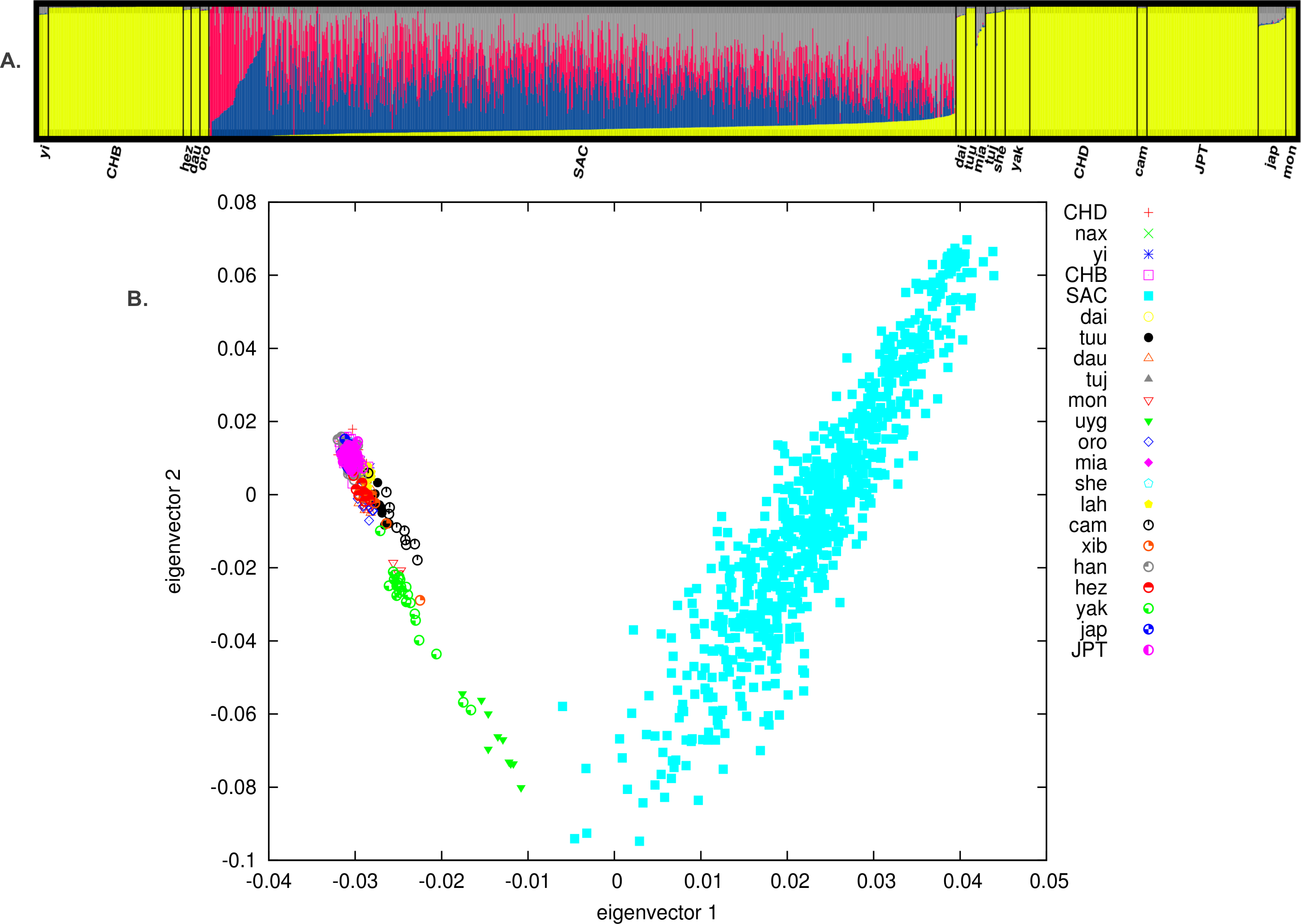

Supplement: Figure S6 — Ancestral population clustering (A) and Principal Component Analysis (B) of the SAC and East Asian populations. (A) The plot in (A) is the proportion of each individual’s ancestry. (B) The plot is of the first and the second eigenvectors in the PCA of the combined populations. For clarity, the population labels in figure (A) are ordered as yi, CHB, hez, dau, oro, SAC, dai, tuu, mia, tuj, she, yak, CHD, cam, JPT, jap and mon (Table S1). (TIFF) [file pone.0073971.s006.tiff]

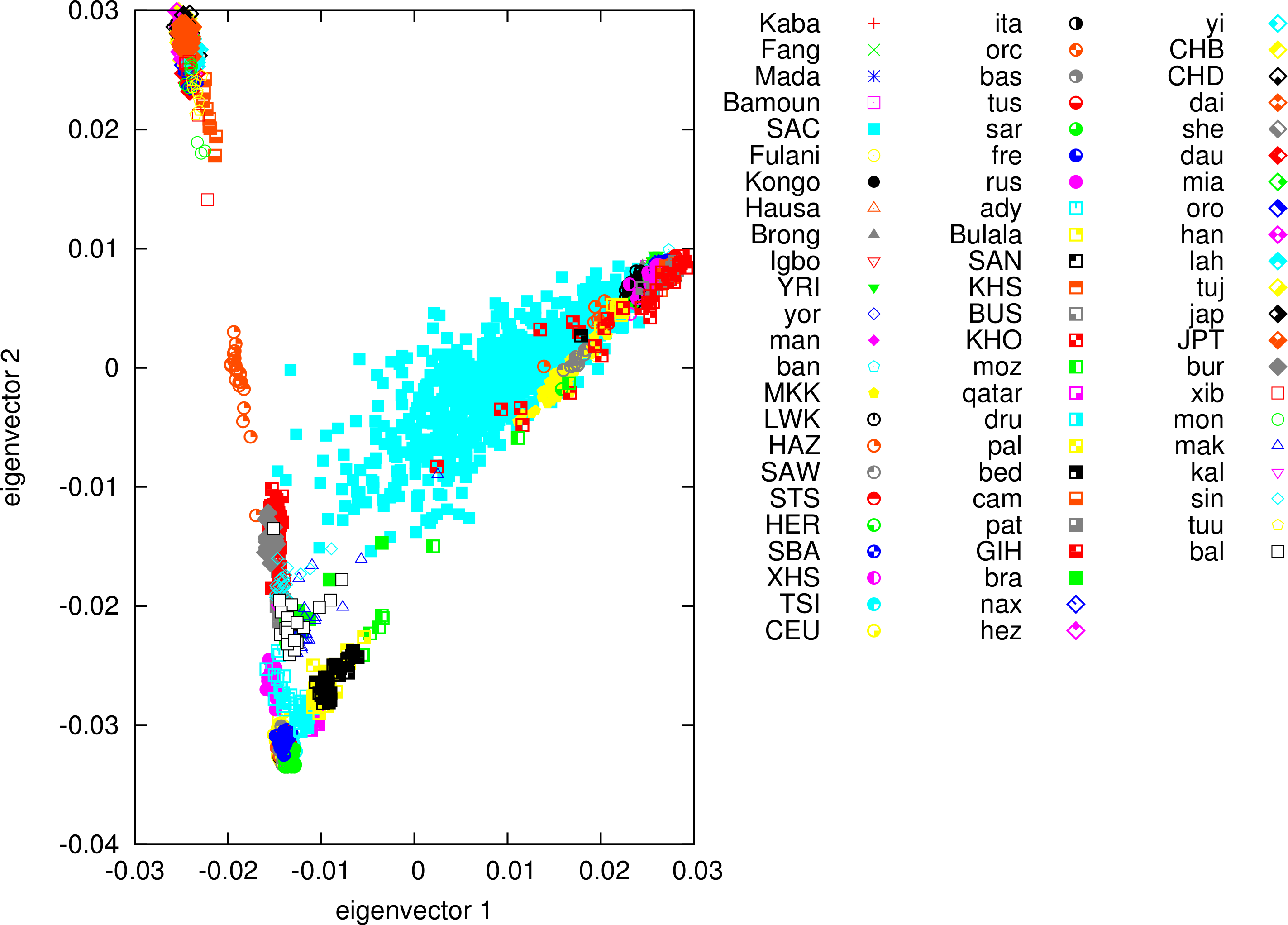

Supplement: Figure S7 — Principal Component Analysis (PCA) of the SAC and the World-wide populations (Table S1). The first and the second eigenvectors in the PCA of the combined SAC and worldwide populations are shown. (TIFF) [file pone.0073971.s007.tiff]

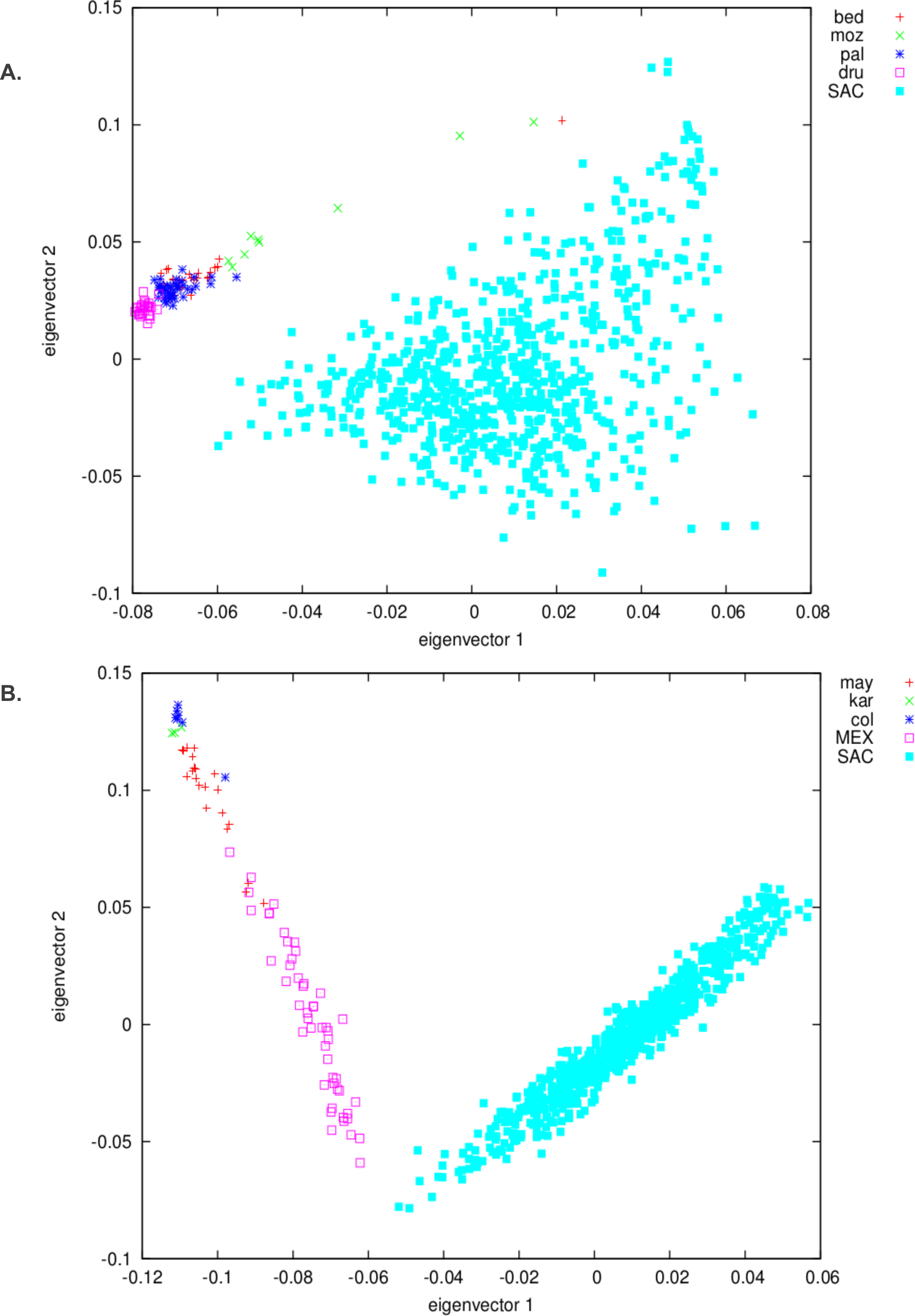

Supplement: Figure S8 — Principal Component Analysis of the SAC and both American and Middle-east populations, respectively. (Table S1). (A) The first and the second eigenvectors in the PCA of the combined SAC and American populations. (B) The first and the second eigenvectors in the PCA of the combined SAC and Middle-east populations. Both figures in (A) and (B) show no evidence of relatedness between the SAC and populations from America and Middle-east. (TIFF) [file pone.0073971.s008.tiff]

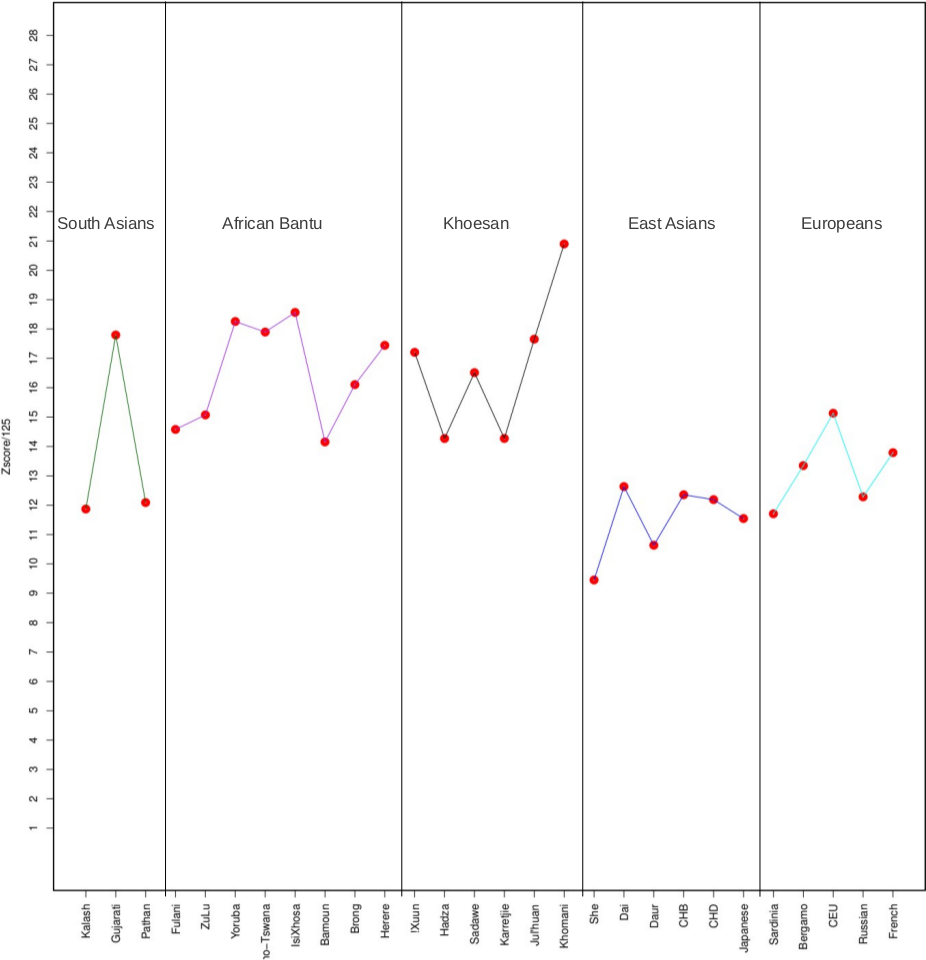

Supplement: Figure S9 — Plot of proxy-ancestry scores (Subjects and Methods) of each population in each group of reference populations. The highest peaks indicates the best proxy ancestry for the South African Coloured population. (TIFF) [file pone.0073971.s009.tiff]

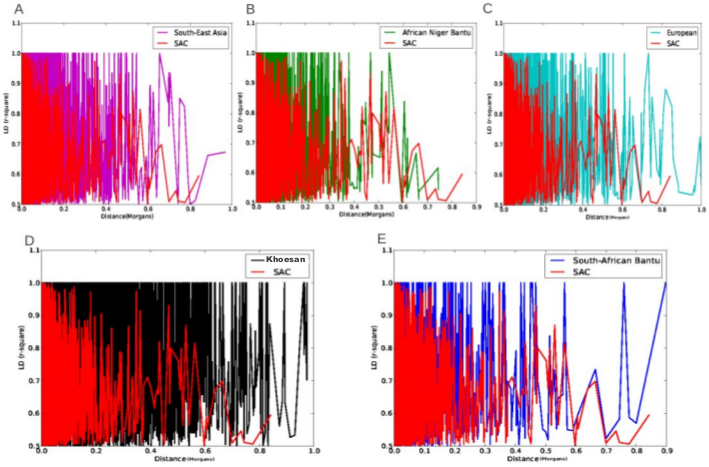

Supplement: Figure S10 — LD across all the autosomes in the SAC compared with proxy ancestral groups. (A–E). Plots of R-square () between pairs of SNPs (combined linked and unlinked SNPs) within 10 Kb from each other. In the figure, we denote ‡Khomani, CEU, CHD+Gujarati Indian, IsiXhosa and Yoruba as Khoesan, European, South-East Asian, South-African Bantu and African Niger Bantu populations, respectively. (TIFF) [file pone.0073971.s010.tiff]

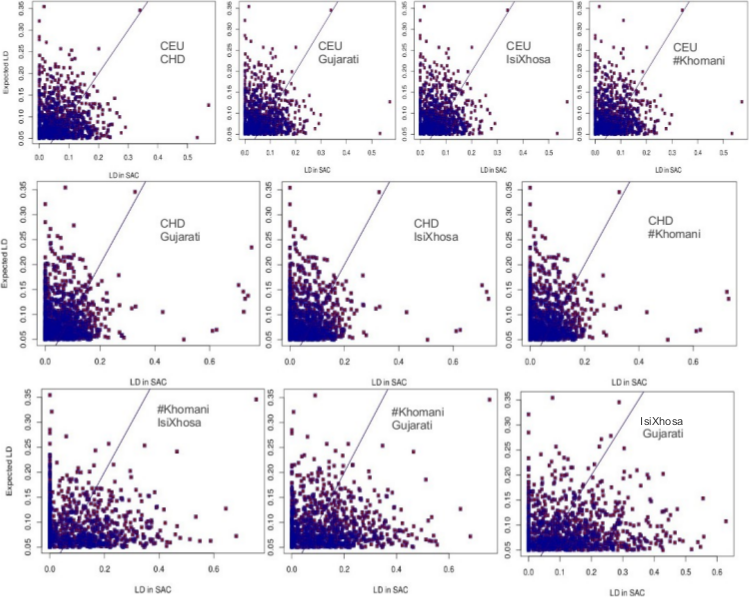

Supplement: Figure S11 — LD due to proxy ancestral population (CEU, ‡Khomani, CHD, Gujarati and IsiXhosa) admixture in the SAC. To generate these plots, we computed the LD between all pairs of markers in the SAC and the expected admixture from each pair of ancestral populations. The figure is the scatter plots of LD in the SAC and the expected admixture LD in pairs of ancestral populations. (TIFF) [file pone.0073971.s011.tiff]
