# Supplementary material for: Determining Ancestry Proportions in Complex Admixture Scenarios in South Africa Using a Novel Proxy Ancestry Selection Method
Source: PLoS One. 2013 Sep 16;8(9):e73971. doi: 10.1371/journal.pone.0073971 (PMC3774743; doi:10.1371/journal.pone.0073971)
Supplement: Table S1 — Putative ancestral populations included in the fine characterization of the South African Coloureds (SAC) and simulation data. (PDF) [file pone.0073971.s012.pdf]

Table S1. Putative ancestral populations included in the fine characterization of the South African Coloureds (SAC) and simulation data.

Pop. Label	Source	Pop Location	Individuals
Admixed South African Coloured			
sac	[1]	South Africa Coloured population	764
African: non Click-speaker			
moz	HGDP	Mozabite, Algeria	9
yor	HGDP	Yoruba in Ibadan Nigeria	21
man	HGDP	Mandenka, Senegal	24
bpg	HGDP	Biaka Pygmy Central Africa	21
mpg	HGDP	Mbuti Pygmy Congo	12
Kaba	HGDP	North of the Central African	17
Fang	HGDP	Equatorial Bantu	15
Fulani	HGDP	West central Africa	2
Bulala	HGDP	Central Chad	12
Mada	HGDP	Cameroon	12
Mausa	HGDP	West Africa Niger and Nigeria	12
Bamoun	HGDP	Cameroon	18
Kongo	HGDP	Atlantic coast of Congo	9
Brong	HGDP	Ghana	8
LWK	HapMap3	Luhya in Webuye, Kenya	104
MKK	HapMap3	Maasai in Kinyawe, Kenya	108
YRI	HapMap3	Yoruba in Ibadan, Nigeria	147
Igbo	[2]	Southeastern Nigeria	15
man	HapMaP3	Mandenka from Africa	22
African: Local South African Populations			
SAN	HGDP	Ju|'hoan, Namibia	5
KHS	[3]	North and South Ju|'hoan, Namibia and Botswana	25
KHO	[2]	‡Khomani, South Africa	8
SBAN	[4]	Bantu-South-Africa	17
STS	[3, 4]	Tswana Southern Africa	16
XHS	[5]	Xhosa, South-Africa	20
XHS2	HGDP	Xhosa, South-Africa	5
HER	[4]	Herero, South Africa and Namibia	14
HAZ	[6]	Hadza,Tanzania	17
BUS	[6]	Bushmen, South Africa	16
SAW	[6]	Sandawe,Tanzania	16
African: Afroasiatic			
ALG	[6]	Algeria	19

Continued on next page

Table 1 – continued from previous page

**Pop. Label**	**Source**	**Pop Location**	**Individuals**
MRC_*N*	[6]	North Morocco	18
LBY	[6]	Libya	17
MRC_*S*	[6]	South Morocco	16
moz	[6] and HGDP	Mozabite	29
EGT	[6]	Egypt	19
SAH_*occ*	[6]	Sahara Occidental	18
TNS	[6]	Berber from Tunisia	18
European			
bas	HGDP	Basque, France	24
sar	HGDP	Sardinian, Italy	27
ita	HGDP	Italian, Italy-Bergamo	13
orc	HGDP	Orkney, Islands	14
fre	HGDP	French, France	29
ady	HGDP	Adygei, Russia-Caucasus	15
rus	HGDP	Russian, Russia	24
TSI	HapMap3	Toscani in Italia	100
CEU	HapMap3	Northern European	112
East Asia			
mia	HGDP	Miao-China	10
jap	HGDP	Japanese, Japan	28
nax	HGDP	Naxi, China	9
dai	HGDP	Dai, China	10
yi	HGDP	Yi, China	10
tuj	HGDP	Tujia, China	10
she	HGDP	She, China	10
lah	HGDP	Lahu, China	7
oro	HGDP	Oroqen, China	10
uyg	HGDP	Uygur, China	9
hez	HGDP	Hezhen, China	9
dau	HGDP	Daur, China	9
xib	HGDP	Xibo, China	9
tuu	HGDP	Tu, China	10
mon	HGDP	Mongola, China	10
cam	HGDP	Cambodian, Cambodia	11
CHB	HapMap3	Han, Chinese in Beijing	137
CHD	HapMap3	Chinese in Denver,Colorado	109
JPT	HapMap3	Japanese in Tokyo	113
South Asia			

Continued on next page

Table 1 – continued from previous page

Pop. Label	Source	Pop Location	Individuals
han	HGDP	Han, Chinese	43
bra	HGDP	Brahui, Pakistan	23
bal	HGDP	Balochi, Pakistan	23
mak	HGDP	Makrani, Pakistan	22
kal	HGDP	Kalash, Pakistan	25
pat	HGDP	Pathan, Pakistan	23
sin	HGDP	Sindhi, Pakistan	25
bur	HGDP	Burusho, Pakistan	23
haz	HGDP	Hazara, Pakistan	22
GIH	HapMap3	Gujarati Indians in Texas	93
		Middle East	
bed	HGDP	Bedouin, Israel-Negev	35
dru	HGDP	Druze, Israel-Carmel	26
yak	HGDP	Yakut, Siberia	19
qatari	[2]	Qatar	22
pal	HGDP	Palestinian, Israel-Central	40

References

1. deWit E, Delport W, Chimusa R, Meintjes A, Moller M, et al. (2010) Genome-wide analysis of the structure of the south African Coloured population in the western Cape. Hum Genet 128, 15-53 .

2. Henn B, Botigue L, Gravel S, Wang W, Brisbin A, et al. (2012) Genomic ancestry of north Africans supports back-to-Africa migrations. Nat Comm 3 (1143) 2140 .

3. Pickrell K, Patterson N, Barbieri C, Berthold F, Gerlach L, et al. (2012) The genetic prehistory of southern Africa. Nature Communications 3 (1143) doi:101038/ncomms2140 .

4. Schlebusch C, Skoglund P, Sjodin P, Gattepaille L, Hernandez D, et al. (2012) Genomic variation in seven Khoe-San groups reveals adaptation and complex African history. Science 338, 374-379 .

5. Chimusa E, Meintjes A, Tchanga M, Mulder N, Soodyall H, et al. (2013) Genome-wide haplotype and signature of selection in indigenous southern African populations. (Preparation) .

6. Henn B, Gignouxb C, Jobinc M, Grankae J, Macphersonf, et al. (2011) Hunter-gatherer genomic diversity suggests a southern African origin for modern humans. PNAS 108, 5154-5162 .
