# Supplementary material for: Determining Ancestry Proportions in Complex Admixture Scenarios in South Africa Using a Novel Proxy Ancestry Selection Method
Source: PLoS One. 2013 Sep 16;8(9):e73971. doi: 10.1371/journal.pone.0073971 (PMC3774743; doi:10.1371/journal.pone.0073971)
Supplement: Table S2 — f3 Statistic: the signal of admixture in the simulation data (simulation obtained from 5-way admixture of ‡Khomani, IsiXhosa, Chinese (CHD) and Indian Gujarati and CEU) using pair-wise ancestral populations. The f3 statistic fails to provide clear evidence/non-evidence of population admixture based on simulated data of a 5-way admixed population. (PDF) [file pone.0073971.s013.pdf]

Table S2. f3 Statistic: the signal of admixture in the simulation data (simulation obtained from 5-way admixture of ‡Khomani, IsiXhosa, Chinese (CHD) and Indian Gujarati and CEU) using pair-wise ancestral populations. The f3 statistic fails to provide clear evidence/non-evidence of population admixture based on simulated data of a 5-way admixed population.

Pop 1	Pop 2	Target	f3	Standard Error	Z
CEU	SAN	Simulated data	−0.008274	0.001486	−5.567
CEU	CHD	Simulated	0.013206	0.000848	15.577
CEU	Dai	Simulated data	−0.007101	0.001613	−4.402
CEU	Daur	Simulated data	−0.00588	0.001473	−3.993
CEU	Druze	Simulated data	−0.004476	0.001406	−3.183
CEU	French	Simulated data	−0.004281	0.001307	−3.275
CEU	Gujarati	Simulated data	0.024759	0.00079	31.328
CEU	Herere	Simulated data	−0.005859	0.001402	−4.178
CEU	IsiXhosa	Simulated data	−0.017475	0.000485	−36.003
CEU	Italian	Simulated data	−0.005691	0.001307	−4.355
CEU	Japanese	Simulated data	−0.005363	0.001526	−3.515
CEU	Ju|'huan	Simulated data	−0.00929	0.00154	−6.034
CEU	Kongo	Simulated data	−0.005008	0.001272	−3.937
CEU	Bushmen	Simulated data	−0.007629	0.001445	−5.281
CEU	‡Khomani	Simulated data	−0.016297	0.000507	−32.134
CEU	Pathan	Simulated data	−0.006022	0.001559	−3.862
CEU	Russian	Simulated data	−0.004508	0.001369	−3.292
CHD	SAN	Simulated data	−0.002889	0.002075	−1.392
CHD	Dai	Simulated data	−0.00351	0.0016	−2.194
CHD	Daur	Simulated data	−0.003448	0.001628	−2.118
CHD	Druze	Simulated data	−0.001977	0.00165	−1.198
CHD	French	Simulated data	−0.000712	0.001629	−0.437
CHD	Gujarati	Simulated data	0.021482	0.000792	27.115
CHD	Herere	Simulated data	−0.00043	0.001972	−0.218
CHD	IsiXhosa	Simulated data	−0.013885	0.000574	−24.189
CHD	Italian	Simulated data	−0.001775	0.001663	−1.067
CHD	Japanese	Simulated data	−0.003516	0.001573	−2.235
CHD	Ju|'huan	Simulated data	−0.004513	0.002272	−1.986
CHD	Kongo	Simulated data	−0.002003	0.001721	−1.161
CHD	Bushmen	Simulated data	−0.003558	0.001973	−1.803
CHD	‡Khomani	Simulated data	−0.011325	0.00058	−19.529
CHD	Pathan	Simulated data	−0.003077	0.001628	−1.889
CHD	Russian	Simulated data	−0.00111	0.001669	−0.665
Gujarati	IsiXhosa	Simulated data	−0.015372	0.00049	−31.344
Gujarati	‡Khomani	Simulated data	−0.014524	0.000514	−28.270
IsiXhosa	SAN	Simulated data	0.012951	0.00108	11.988
IsiXhosa	Dai	Simulated data	0.012467	0.001004	12.414
IsiXhosa	Daur	Simulated data	0.01169	0.001094	10.689
IsiXhosa	Druze	Simulated data	0.011085	0.000988	11.214
IsiXhosa	French	Simulated data	0.010815	0.00096	11.261
IsiXhosa	Herere	Simulated data	0.011161	0.000816	13.675
IsiXhosa	Italian	Simulated data	0.011392	0.000969	11.757
IsiXhosa	Japanese	Simulated data	0.011498	0.001059	10.857
IsiXhosa	Ju|'huan	Simulated data	0.013441	0.001109	12.12
IsiXhosa	Kongo	Simulated data	0.011467	0.000935	12.261
IsiXhosa	Bushmen	Simulated data	0.012622	0.001064	11.859
IsiXhosa	Pathan	Simulated data	0.011657	0.000999	11.672
IsiXhosa	Russian	Simulated data	0.010773	0.000995	10.83
‡Khomani	SAN	Simulated data	0.001143	0.001169	0.978
‡Khomani	Dai	Simulated data	0.00037	0.001085	0.341
‡Khomani	Daur	Simulated data	0.000218	0.001041	0.209
‡Khomani	Druze	Simulated data	−0.001391	0.001057	−1.315
‡Khomani	French	Simulated data	−0.001505	0.000977	−1.54
‡Khomani	Herere	Simulated data	−0.000838	0.001046	−0.801
‡Khomani	IsiXhosa	Simulated data	0.002469	0.000364	6.79
‡Khomani	Italian	Simulated data	−0.00128	0.001029	−1.243
‡Khomani	Japanese	Simulated data	−0.000415	0.001039	−0.399
‡Khomani	Ju|'huan	Simulated data	0.002027	0.00121	1.675
‡Khomani	Kongo	Simulated data	−0.000756	0.000957	−0.79
‡Khomani	Bushmen	Simulated data	0.000484	0.001189	0.407
‡Khomani	Pathan	Simulated data	−0.000232	0.001071	−0.217
‡Khomani	Russian	Simulated data	−0.00108	0.000986	−1.095
