# Supplementary material for: Determining Ancestry Proportions in Complex Admixture Scenarios in South Africa Using a Novel Proxy Ancestry Selection Method
Source: PLoS One. 2013 Sep 16;8(9):e73971. doi: 10.1371/journal.pone.0073971 (PMC3774743; doi:10.1371/journal.pone.0073971)
Supplement: Table S3 — Comparing genetic diversity between the South African Coloured population (SAC) and the five proxy ancestral groups contributing to the SAC admixture. Mean and standard error of shared haplotype segment in cM (Hap.segment), mean and standard error of haplotype diversity measure (Hap.diversity) and proportion of IBD (Prop.IBD). (PDF) [file pone.0073971.s014.pdf]

Table S3. Comparing genetic diversity between the South African Coloured population (SAC) and the five proxy ancestral groups contributing to the SAC admixture. Mean and standard error of shared haplotype segment in cM (Hap. Segment), mean and standard error of haplotype diversity measure (Hap. diversity) and proportion of IBD (Prop. IBD).

	**Hap. Segment**	**Hap. diversity**	**Prop. IBD**
**SAC**	1.022 ± 0.004	81.975 ± 0.002	(0.0018)
**isiXhosa**	0.9058 ± 0.042	16.860 ± 0.003	(0.0284)
**‡Khomani**	1.123 ± 0.033	5.214 ± 0.004	(0.1714)
**CEU**	1.192 ± 0.043	50.544 ± 0.003	(0.0189)
**CHD**	0.715 ± 0.0417	54.885 ± 0.003	(0.1051)
**Gujarati**	0.614 ± 0.042	57.883 ± 0.003	(0.0512)
