# Supplementary material for: Determining Ancestry Proportions in Complex Admixture Scenarios in South Africa Using a Novel Proxy Ancestry Selection Method
Source: PLoS One. 2013 Sep 16;8(9):e73971. doi: 10.1371/journal.pone.0073971 (PMC3774743; doi:10.1371/journal.pone.0073971)
Supplement: File S1 — PROXYANC: -optimal Quadratic Cone Programming. (PDF) [file pone.0073971.s015.pdf]

PROXYANC: F_{ST}-optimal Quadratic Cone Programming

To limit the effect of background linkage disequilibrium, we assume adjacent SNPs in each population that are spaced 10 Kb from each other. Let Z denote a set of pools of distinct reference ancestral populations. Suppose we have SNP j, let N_j and p_j be the total variant allele count and observed population allele-frequency in the admixed population (A), and N_{jk} and p_{jk} be the total variant allele count and the population observed allele-frequency in reference population $k = 1, 2, \dots, K$ of unrelated individuals. Given different combinations C of $L = |Z|$ reference populations of unrelated individuals from each pool $S_i \in Z = \mathbb{N}^L, (i = 1, \dots, L)$, each combination C of L reference populations can be obtained from the Cartesian product $T = \prod_{i=1}^{L} S_i, C \in Z$. Thus, from each $C \in Z$ we construct synthetic populations consisting of L populations as the following linear combination,

$$p_{j\alpha} = \sum_{k=1}^{L} \alpha_l p_{jl}, \tag{1}$$

where α_l is the ancestral proportion. A particular combination of L populations (synthetic admixed population) consists of the best proxy ancestries of A if their linear combination (in equation 1) minimizes the constructed objective (in equation 2) function $\tilde{F}_j \approx F_{ST}(A, p_{j\alpha})$. $\tilde{F}_j$ is approximated from a classical F_{ST} function in order to render the optimization problem convex. This problem is related to optimal quadratic cone programming, where the objective function $\tilde{F}_j$ is given at each SNP j by,

$$\tilde{F}_j(\alpha) = \left[(p_{j\alpha} - p_j)^2 - p_j \frac{(1-p_j)}{N_j} - \sum_{l=1}^{L} \alpha_l^2 p_j \frac{(1-p_j)}{N_{jl}} \right] \times \frac{1}{p_j(1-p_j).L}, \tag{2}$$

subject to $\sum_{l=1}^{L} \alpha_l = 1$ and

$$\alpha_l \leqslant 0,\ \forall l \in \{1, \dots, L\}.$$

Equation 2 is a generalization of the objective function described in [1], and is a quadratic convex function with respect to α_l (ancestry proportion), therefore a global minimum can be found. To obtain a matrix representation of the optimal cone programming, equation 2 can be expanded. Let us denote $C_1 = \frac{1}{p_j(1-p_j)K}$, $C_2 = p_j(1-p_j)$, and $C_3 = p_j \frac{(1-p_j)}{N_j}$. Thus, equation 2 becomes,

$$\tilde{F}_j(\alpha) = \left[(p_{j\alpha} - p_j)^2 - C_3 - \sum_{l=1}^{L} \frac{\alpha_l^2}{N_{jl}} C_2 \right] \times C_1. \tag{3}$$

It follows that,

$$\tilde{F}_j(\alpha) = \left[p_{j\alpha}^2 - 2p_{j\alpha}p_j + \underbrace{p_j^2 - C_3}_{C_4} - \sum_{l=1}^{L} \frac{\alpha_l^2}{N_{jl}} C_2 \right] \times C_1. \tag{4}$$

Substituting equation 1 into equation 4, we obtain,

$$\tilde{F}_j(\alpha) = \left[(\sum_{l=1}^{L} \alpha_l p_{jk})^2 - 2\sum_{l=1}^{L} \alpha_l p_{jl} p_j + C_4 - \sum_{l=1}^{L} \frac{\alpha_l^2}{N_{jl}} C_2 \right] \times C_1. \tag{5}$$

Now expanding equation 5, using a squared finite sum,

$$(\sum_{l=0}^{L} x_l)^2 = \sum_{l=0}^{L} x_l^2 + \sum_{l \neq n} x_l x_n,$$

such that x is a variable, it follows that

$$\begin{aligned}\tilde{F}_j(\alpha) &= \left[\sum_{l=1}^{L}\alpha_l^2 p_{jk}^2 + \sum_{l\neq n}(\alpha_l\alpha_n)p_{jl}p_{jn} - 2\sum_{l=1}^{L}\alpha_l p_{jl}p_j + C_4 - \sum_{l=1}^{L}\frac{\alpha_l^2}{N_{jl}}C_2\right]\times C_1\\ &= \left[\sum_{k=1}^{L}\alpha_l^2(p_{jl}^2 - \frac{C_2}{N_{jl}}) + \sum_{l\neq n}(\alpha_l\alpha_n)p_{jl}p_{jn} - 2\sum_{l=1}^{L}\alpha_l p_{jl}p_j + C_4\right]\times C_1. \end{aligned} \tag{6}$$

Knowing that the ancestral proportion must sum to 1, $\sum_{l=1}^{L}\alpha_l = 1$ then

$$\sum_{l=1}^{L}\alpha_l C_4 = C_4,$$

and equation 6 becomes,

$$\begin{aligned}\tilde{F}_j(\alpha) &= \left[\sum_{l=1}^{L}\alpha_l^2(p_{jl}^2 - \frac{C_2}{N_{jl}})C_1\right] + \left[\sum_{l\neq n}(\alpha_l\alpha_n)p_{jl}p_{jn}C_1\right] - 2\sum_{l=1}^{L}\alpha_l p_{jl}p_j C_1 + \sum_{l=1}^{L}\alpha_l C_4 C_1\\ &= \left[\sum_{l=1}^{L}\alpha_l^2(p_{jl}^2 - \frac{C_2}{N_{jl}})C_1\right] + \left[\sum_{l\neq n}(\alpha_l\alpha_n)p_{jl}p_{jn}C_1\right] + \left[\sum_{l=1}^{L}\alpha_l(C_4 - 2p_{jl}p_j)C_1\right]. \end{aligned} \tag{7}$$

Therefore, the matrice representation of the optimal Cone Programming can be obtained as follows,

$$min_\alpha = \left(\frac{1}{2}\alpha^T P\alpha + q^T\alpha\right)\text{subject to } -\alpha G \leqslant 0 \text{ and } \alpha A = 1, \tag{8}$$

where α is a vector of L-dimensions of unknown ancestry proportions, G is an identity vector of L-dimensions, A is a vector of allele frequencies of L-dimensions, P is a positive semi definite matrice, and its diagonal elements are all coefficients of α^2:

$$(\alpha^2)_l = 2\frac{p_{jl}^2 - \frac{p_j(1-p_j)}{N_{jl}}}{p_j(1-p_j)L}, \tag{9}$$

and the mixture coefficients $\alpha_l\alpha_n$ consist of its symmetric elements, and are given by:

$$(\alpha)_{ln} = 2\frac{p_{jl}p_{jn}}{p_j(1-p_j)L}, \qquad \text{for } k \neq n, \tag{10}$$

and the linear coefficients α_l are the elements of vector q in equation 8, and are represented by:

$$(\alpha)_l = \frac{(p_j^2 - p_j\frac{(1-p_j)}{N_j} - 2p_{jl}p_j)}{p_j(1-p_j)L}. \tag{11}$$

For the optimization of equations (3) or (2) with respect to α_l (ancestry proportions, $l = 1, \ldots, L$), the matrix form in equation (3) is constructed by summing equations (2), (4), (5) and (6) independently across all SNPs.

References

1. Price A, Helgason A, Palsson S, Stefansson H, Clair D, et al. (2009) The impact of divergence time on the nature of population structure: An example from Iceland. PLoS Genet 5(6), e1000505 .
